# Orbitofrontal Thickness and Network Associations as Transdiagnostic Signature of Amotivation Along the Bipolar-Schizophrenia Spectrum

**DOI:** 10.1093/schbul/sbaf078

**Published:** 2025-06-20

**Authors:** Marlene Franz, Valeria Kebets, Xaver Berg, Foivos Georgiadis, Beatrice A Milano, Achim Burrer, Janis Brakowski, Stefan Kaiser, Erich Seifritz, Philipp Homan, Esther Walton, Theo G M van Erp, Jessica A Turner, Bratislav Misic, Sofie L Valk, B T Thomas Yeo, Boris C Bernhardt, Matthias Kirschner

**Affiliations:** Department of Psychiatry, Psychotherapy and Psychosomatics, Psychiatric Hospital University of Zurich, 8032 Zurich, Switzerland; McConnell Brain Imaging Centre, Montreal Neurological Institute and Hospital, McGill University, 3801 Montreal, Quebec, Canada; Department of Psychiatry, Psychotherapy and Psychosomatics, Psychiatric Hospital University of Zurich, 8032 Zurich, Switzerland; Department of Psychiatry, Psychotherapy and Psychosomatics, Psychiatric Hospital University of Zurich, 8032 Zurich, Switzerland; Division of Adult Psychiatry, Department of Psychiatry, Geneva University Hospitals, 1226 Thônex, Switzerland; Department of Psychiatry, Psychotherapy and Psychosomatics, Psychiatric Hospital University of Zurich, 8032 Zurich, Switzerland; Department of Psychiatry, Psychotherapy and Psychosomatics, Psychiatric Hospital University of Zurich, 8032 Zurich, Switzerland; Division of Adult Psychiatry, Department of Psychiatry, Geneva University Hospitals, 1226 Thônex, Switzerland; Department of Psychiatry, Psychotherapy and Psychosomatics, Psychiatric Hospital University of Zurich, 8032 Zurich, Switzerland; Department of Psychiatry, Psychotherapy and Psychosomatics, Psychiatric Hospital University of Zurich, 8032 Zurich, Switzerland; Neuroscience Center Zurich, University of Zurich and Swiss Federal Institute of Technology Zurich, Switzerland; Department of Psychology, University of Bath, BA2 7AY Bath, United Kingdom; Clinical Translational Neuroscience Laboratory, Department of Psychiatry and Human Behavior, University of California Irvine, 92697 Irvine, CA, United States; Center for the Neurobiology of Learning and Memory, University of California Irvine, 92697 Irvine, CA, United States; Department of Psychiatry and Behavioral Health, The Ohio State University, Columbus, OH 43210, United States; McConnell Brain Imaging Centre, Montreal Neurological Institute and Hospital, McGill University, 3801 Montreal, Quebec, Canada; Institute for Neuroscience and Medicine (INM-7), Forschungszentrum Jülich, 52425 Jülich, Germany; Max Planck Institute for Human Cognitive and Brain Sciences, 04103 Leipzig, Germany; Max Planck School of Cognition, 04103 Leipzig, Germany; Institute of Systems Neuroscience, Medical Faculty and University Hospital Düsseldorf, Heinrich Heine University Düsseldorf, 40225 Düsseldorf, Germany; Centre for Sleep & Cognition, Centre for Translational MR Research, Department of Electrical & Computer Engineering, Department of Medicine, National University of Singapore, 117549 Singapore, Singapore; McConnell Brain Imaging Centre, Montreal Neurological Institute and Hospital, McGill University, 3801 Montreal, Quebec, Canada; Department of Psychiatry, Psychotherapy and Psychosomatics, Psychiatric Hospital University of Zurich, 8032 Zurich, Switzerland; Division of Adult Psychiatry, Department of Psychiatry, Geneva University Hospitals, 1226 Thônex, Switzerland

**Keywords:** negative symptoms, amotivation, diminished expression, orbitofrontal cortex, MRI, structural covariance, epicenter mapping, schizophrenia, bipolar disorder

## Abstract

**Background and Hypothesis:**

Negative symptoms of schizophrenia (SCZ), particularly amotivation, are prominent across both SCZ and bipolar disorder (BD). While orbitofrontal cortex (OFC) alterations have been implicated in the development of negative symptoms, their contributions across disorders remain to be established. Here, we examined how OFC thickness and network associations relate to amotivation compared to diminished expression across the BD-SCZ spectrum.

**Study Design:**

We included 50 individuals with SCZ, 49 with BD, and 122 controls. We assessed amotivation and diminished expression and estimated thickness in the medial and lateral OFC as regions of interest as well as 64 other cortical regions.

**Study Results:**

Across BD and SCZ, reduced right lateral and bilateral medial OFC thickness were specifically associated with amotivation, but not diminished expression or other clinical factors. We then generated intra-individual OFC structural covariance networks to evaluate how the system-level embedding of the OFC would link to brain-wide cortical maps of negative symptoms. We found that medial OFC covariance networks spatially correlated with the brain-wide cortical alterations of both negative symptom dimensions. Further analyses in independent SCZ data from the ENIGMA consortium (*n* = 4474) revealed associations with lateral OFC covariance networks. Finally, the brain-wide cortical alterations of amotivation were significantly correlated with normative functional and structural white-matter connectivity profiles of the right medial and left lateral OFC as well as adjacent prefrontal and limbic regions.

**Conclusions:**

Our work identifies OFC alterations as a possible transdiagnostic signature of amotivation and provides insights into network associations underlying the system-wide cortical alterations of negative symptoms across SCZ and BD.

## Introduction

Negative symptoms in schizophrenia (SCZ) significantly affect clinical outcomes, with avolition, anhedonia, and asociality clustering as the amotivation dimension, while alogia and blunted effect constitute the diminished expression dimension.^1–10^ Both negative symptom dimensions are differentially associated with functioning and cognition^1,2,11,12^ suggesting distinct, albeit interconnected, underlying neural pathways.^13^ Amotivation has been linked to reward-processing circuits, including the OFC and striatum,^8,13–15^ whereas diminished expression may more strongly involve socio-affective processing networks including prefrontal, limbic, and temporal regions.^13,16–21^ Furthermore, the amotivation dimension is now recognized as a transdiagnostic feature across the bipolar disorder (BD)-SCZ spectrum,^22–25^ and has been related to neurocognitive impairments in both SCZ and BD,^26,27^ raising the possibility of a shared neural basis.

On a neural level, amotivation in SCZ is linked to prefrontal-striatal reward network alterations^13–15^ including altered prefrontal-striatal reward signals and connectivity.^28–36^ In line with this prefrontal-striatal dysfunction hypothesis, neuroanatomical alterations within these regions have also been associated with higher amotivation across neurological disorders and SCZ.^37,38^ However, while functional neuroimaging studies highlight altered striatal activity during reward processing to negative symptoms in SCZ,^29–35,39^ morphometric findings on striatal subregions have been inconsistent, with studies reporting negative,^40,41^ positive,^42^ or null associations in SCZ.^43,44^ In contrast, orbitofrontal cortex (OFC) thickness alterations have emerged as a robust neural signature of negative symptoms, particularly amotivation, across disease stages in SCZ.^42,43,45–49^ For example, a meta-analysis from the ENIGMA consortium found that reduced lateral and medial OFC thickness is correlated with higher negative symptoms in SCZ.^48^ Recent work from our group and others extended these findings showing that these associations are also detectable in individuals with first-episode psychosis.^43,47,49^

However, despite growing evidence linking neuroanatomical OFC alterations to negative symptoms in SZ, its transdiagnostic role across the BD-SCZ spectrum remains unclear. Investigations into the brain signatures of common symptom dimensions across BD and SCZ are supported by evidence of overlapping morphometric alterations associated with these disorders. Recent meta-analyses and cross-disorder studies have identified shared OFC thickness reductions with comparable effect sizes.^50–52^ It has been shown that the observed patterns of cortical abnormalities reflect covariance networks, such that regions exhibiting more similar cortical thickness variations tend to be more strongly connected to each other.^53,54^ In this regard, recent work identified associations between regional structural covariance networks and distinct symptom dimensions in BD and SCZ.^55,56^ These results support the hypothesis that brain-wide symptom–structure relationships might be guided by localized regions, which in turn have strong associations with the respective symptom. In addition, it has been shown that disease-specific cortical alterations of BD and SCZ are constrained by overlapping normative functional and structural connectome features.^57,58^ This comparison of cortical alterations to normative regional functional and structural connectivity profiles, defined as epicenter mapping, has been initially developed to identify epicenters of atrophy spread in neurodegenerative disorders.^59–61^ More recently this epicenter mapping has been validated in several studies examining disease-specific and cross-disorder epicenters in neuropsychiatric disorders. These findings support the hypothesis that disease- or symptom-specific cortical alteration patterns may propagate from distinct epicenters to other cortical regions via network mechanisms.^53,57,62–64^ Together these findings suggest that the connectivity profile of specific brain regions shapes the disease or symptom-specific spatial pattern of cortical alterations. Based on this complementary research on the network characteristics of cortical alterations in BD and SCZ, 2 research questions arise. First, does the system-level embedding of OFC, as measured by OFC structural covariance networks, align with the brain-wide cortical alterations associated with amotivation? Second, do functional or structural cortico-cortical connectivity profiles of the OFC or adjacent regions spatially correlate with the brain-wide cortical alterations associated with amotivation, indicating network propagation from putative epicenters?

Integrating multiple lines of research, our study investigates whether localized OFC thickness alterations and OFC covariance networks constitute transdiagnostic signatures of amotivation across the BD-SCZ spectrum. In addition, we assess whether the brain-wide cortical alterations associated with amotivation are constrained by normative functional and structural connectivity, providing insight into its potential network-level propagation. Leveraging structural imaging data from individuals with BD and SCZ, we hypothesized that reduced OFC thickness correlates with higher negative symptoms, particularly amotivation, independent of other clinical factors. We further hypothesized that cortical regions whose thickness more strongly covaries with OFC thickness would also show stronger associations with amotivation. Finally, we hypothesized that the brain-wide cortical alterations associated with amotivation align with the normative functional and structural connectivity of the OFC, which may reflect network-level constraints on symptom-related cortical alterations.

## Methods

### Participants

Clinical and structural imaging data were obtained from the UCLA CNP cohort (University of California, Consortium for Neuropsychiatric Phenomics) and downloaded from the public database OpenfMRI (https://openfmri.org/dataset/ds000030/). Data from 50 patients diagnosed with SCZ and 49 patients diagnosed with BD type I were included in the main analysis of this study. In addition, 122 healthy controls (HCs) from the same UCLA CNP cohort were included for additional group comparisons (for details see Supplementary Methods and Results). All participants were part of a larger multimodal imaging study which has been described in detail elsewhere.^65^ In brief, all participants were recruited from the Los Angeles area and included if the following inclusion criteria were met: age 21-50 years; primary language either English or Spanish; at least 8 years of formal education; no significant medical illness; negative drug test. HC participants were excluded if they had a lifetime diagnosis of a psychiatric disorder, substance abuse or dependence. BD type I participants were not allowed to have a diagnosis of SCZ and vice versa. All participants with BD type I or SCZ were outpatient individuals presenting clinical stability to complete the clinical assessment and extensive multimodal imaging sessions. Diagnoses were based on DSM-IV criteria using a semi-structured assessment with Structured Clinical Interview (SCID) for the DSM-IV. For details, see the study by Zhou et al. (2012).^65^

## Image Acquisition and Processing

T1-weighted structural brain scans were processed using FreeSurfer Version 5.3.0^66^ and were derived from a previous resting-state fMRI publication on the same dataset.^67^ We extracted cortical thickness for 68 Desikan-Killiany (DK) atlas regions^68^ (right and left hemisphere; 34 regions per hemisphere) as well as subcortical volume (SV) from 6 bilateral striatal subregions (nucleus accumbens, caudate, and putamen). We performed quality control (QC) using standard ENIGMA protocol (http://enigma.ini.usc.edu/protocols/imaging-protocols).^52^ After QC, 4 participants (HC = 3, SCZ = 1) were excluded due to poor segmentation resulting in a final sample of 49 patients with SCZ, 49 patients with BD, and 119 controls. To account for scanner and site effects data were harmonized using ComBat.^69^ Finally, the cortical thickness of each region was corrected for age and sex and the standardized residuals were used for all subsequent analyses. To address our a priori hypotheses, we applied a region of interest (ROI) approach focusing on the standardized residuals of left and right lateral OFC and medial OFC thickness (total ROIs = 4) (Supplementary Table S1).

## Assessment of Negative Symptoms and Other Clinical Factors

Negative symptom severity was assessed with the Scale for the Assessment of Negative Symptoms^70^ (SANS) using the global scores of the negative symptom domains blunted effect, alogia, avolition-apathy, and anhedonia-asociality. To measure the 2 negative symptom dimensions of amotivation and diminished expression, we followed the approach of previous studies, including our own work on the relationship between cortico-subcortical alterations and negative symptoms across stages of the schizophrenia spectrum.^4,43^ The amotivation dimension was defined by the sum of the avolition-apathy and anhedonia-asociality global scores and the diminished expression dimension by the sum of the blunted affect and alogia global scores.

In addition to negative symptoms, positive, depressive, and disorganized symptoms as well as risperidone equivalent dose (mg/d) were calculated as potential secondary sources of negative symptoms.^28,71^ To assess the severity of positive symptoms, the Scale for the Assessment of Positive Symptoms (SAPS) was used.^70^ The SAPS includes the 4 positive symptom domains delusion, hallucination, bizarre behavior, and positive formal thought. Following previous work,^72,73^ the delusion and hallucination domains were combined to form the SAPS positive symptoms dimension, while the bizarre behavior and positive formal thought domains were combined to form the SAPS disorganization dimension. Depressive symptoms were assessed using the Hamilton Depression Scale with 21 items (HAMD 21).^74^ Risperidone equivalent dose was determined by converting doses of individual antipsychotics using the defined daily doses (DDD) method.^75^

## Statistical Analysis

Analyses were performed using SPSS Version 28.0.1.1(14) (IBM SPSS Inc.), Matlab Version 9.12.0 (R2022a), and R (2009-2021 RStudio, PBS; 2021,09.0 Build 351).

## Group Comparison of OFC Thickness

Prior to our main analyses of assessing the relationship between OFC thickness and amotivation across BD and SCZ, we compared the left/right lateral and medial OFC residuals (*n* = 4) between HC, BD, and SCZ. Using separate analysis of variance (ANOVA), we did not find any differences in OFC thickness between BD and SCZ. For details see Supplementary Table S2.

## Overview of Main Analysis

**Figure 1.**
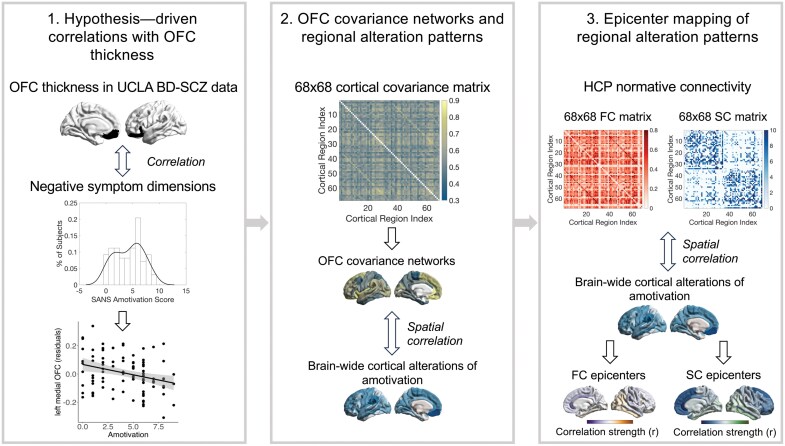
Schematic Figure of Analytical Approach. (1) For Simplicity Correlations are Only Shown for the Amotivation Dimension But have Also been Conducted with the Diminished Expression Dimension and Other Clinical Factors. (2) Please Note That This Analysis was Conducted with the UCLA BD-SCZ Spectrum Sample as well as an Independent Meta-analytic SZ Sample Derived from the ENIGMA Consortium.^52^ Spatial Correlation Refers to the Statistical Approach Described by Alexander-Bloch et al.^76^ FC, functional connectivity, HCP. Human Connectome Project^77,78^ normative connectivity data derived from the ENIGMA toolbox,^79^ SC, structural connectivity, UCLA, UCLA CNP cohort (University of California, Consortium for Neuropsychiatric Phenomics) and downloaded from the public database OpenfMRI (https://openfmri.org/dataset/ds000030/).

Figure 1 outlines the 3 levels of the main analysis: (1) Hypothesis-driven correlations between OFC thickness and amotivation compared to diminished expression; (2) association between OFC covariance networks and brain-wide cortical alterations associated with both negative symptom dimensions; and (3) epicenter mapping of brain-wide cortical alterations of both negative symptom dimension.

## Relationship between OFC Thickness and Amotivation

Spearman`s rank correlations were used to investigate the associations between SANS amotivation scores and the residuals of left/right lateral and medial OFC across all individuals with SCZ and BD. To validate the stability of these associations, we applied bootstrap resampling and generated 1000 samples with replacement of the bilateral lateral and medial OFC variables. We then calculated the 95% confidence intervals by correlating the original SANS amotivation scores with each of the derived bootstrap samples. We further aimed to establish whether associations with lateral and medial OFC thickness reflect specific neural substrates of amotivation or are also related to the diminished expression dimension. We repeated the correlation analyses with the diminished expression dimension and compared differences in the magnitude of the correlation coefficient of the different negative symptom dimensions using Hittner et al.’s method for nonparametric correlations.^80,81^ All correlations between the 4 OFC ROIs and the 2 negative symptom dimensions were corrected for multiple comparisons using a false discovery rate (FDR) of *q* < 0.05. Separate confirmatory correlation analyses in SCZ and BD tested whether transdiagnostic relationships between amotivation and OFC thickness were also significant in each diagnostic group. As an exploratory analysis, we further examined whether subcortical volumes of the left and right nucleus accumbens, caudate, and putamen (residualized for age, sex, and intracranial volume) were associated with either negative symptom dimension.

## Relationship between OFC Thickness and Other Clinical Factors

We next assessed whether associations between OFC thickness and symptom severity are specific signatures of negative symptoms or are also influenced by other symptom dimensions or medication status. We performed exploratory Spearman rank correlations between the left/right lateral OFC and medial OFC thickness with the SAPS positive symptom and SAPS disorganization dimensions, depressive symptoms (HAMD21), and risperidone equivalent dose (mg/d).

## OFC Covariance Networks and Cortical Alteration of Amotivation

Morphological characteristics co-vary across cortical regions and form distinct cortico-cortical covariance networks.^53,82–84^ Building on these findings, we examined whether cortical regions whose thickness more strongly covaries with medial and lateral OFC thickness would also show stronger associations with amotivation (Figure 1). We estimated the joint cortical variation of each cortical region with each OFC ROI resulting in 4 separate OFC covariance networks. We applied the intra-individual structural covariance approach from Yun and colleagues that calculates the inverse exponential of the difference between brain morphological values.^85^ First, age and sex were regressed out from cortical thickness values of the BD-SCZ sample (*n* = 98) and the resulting residuals were normalized using *z*-score transformation. For each individual, we then estimated the intra-individual joint variation between the *i*th (for *i* = 1-68) and *j*th (for *j* = 1-68) cortical region to be equal to the inverse squared difference between the residuals of the *i*th cortical region and the residuals of the *j*th cortical region.

The derived intra-individual (*n* = 98) regional covariance networks (*n* = 68) were then averaged to calculate mean covariance networks for each cortical region (*n* = 68) across the SCZ and BD spectrum. Next, across the SCZ and BD spectrum (*n* = 98), we correlated the residuals of each cortical region (*n* = 68) with amotivation severity to characterize the brain-wide spatial pattern of cortical alterations associated with amotivation. We then correlated each of the 68 covariance networks with the brain-wide cortical alterations associated with amotivation (reflected by correlation coefficients per cortical region), to test the hypothesis that cortical regions whose thickness covaries more strongly with OFC also show stronger associations with amotivation. We assessed statistical significance by correcting for spatial autocorrelation using a spin test^76,79,86^ (1000 repetitions). The specificity of this association was examined by repeating the analysis using the brain-wide spatial pattern of cortical alterations associated with diminished expression. To further assess the robustness of these findings, we repeated the analysis using independent meta-analytic cortical thickness maps derived from 4474 individuals with schizophrenia from the ENIGMA consortium^52^ (see Supplementary Tables S3-S6). We used the same intra-individual covariance approach as described above and generated OFC covariance networks based on the mean cortical thickness values of the ENIGMA schizophrenia sample (Supplementary Table S6, Supplementary Figure S1). We then compared these OFC covariance networks to the meta-analytic brain-wide cortical alterations associated with total negative symptoms (Supplementary Figure S2).^52^ Please note that these meta-analytic data include only brain-wide cortical alterations of total negative symptom scores (PANSS negative and SANS total score) and did not allow further differentiation into the 2 negative symptom dimensions.^52^

## Epicenter Mapping of Cortical Alteration of Amotivation

Next, we tested whether the region-specific cortico-cortical connectivity profile of the OFC or other cortical regions spatially correlates with the brain-wide cortical alteration pattern associated with amotivation severity (Figure 1). High spatial similarity of a region’s connectivity profile with the amotivation-related cortical alteration pattern would be indicative that this region is a putative epicenter from alterations spread to connected regions.^53,57–59,62^ We applied the epicenter mapping approach implemented in the ENIGMA Toolbox^64^ that uses normative functional and structural connectome data derived from resting-state fMRI, and diffusion MRI of unrelated healthy adults (*n* = 207, 83 males, mean age ± SD = 28.73 ± 3.73 years, range = 22-36 years) from the Human Connectome Project (HCP) (Supplementary Figures S3 and S4).^77,78^ Details on preprocessing and connectivity matrix generation can be found elsewhere.^79^ In brief, we spatially correlated every region’s healthy functional and structural cortico-cortical connectivity profile (derived from HCP) with the brain-wide cortical alterations of amotivation in BD and SCZ. We repeated this approach systematically for each parcellated region with functional and structural cortical connectivity separately. Statistical significance of the spatial similarity between an individual brain region’s functional (or structural) connectivity profile and amotivation-related cortical alterations was determined through spatial correlation, correcting for spatial autocorrelation by using 1000 randomly rotated cortical maps based on the spin test.^76,79^ Regions were ranked in descending order based on the strength of their correlation coefficients, with the highest-ranked regions being considered the most significant disease epicenters. Please note that this definition of epicenters does not require the epicenter region itself to have a strong association with amotivation. Instead, an epicenter is defined by its connectivity strength to regions with high cortical alteration and its weak connections to regions with low cortical alteration.^57,62,64^ In addition, epicenters are not necessarily hub regions with many direct connections but can also include regions that are directly connected to hub regions, so-called feeder nodes, from which network-spreading effects may propagate via their connections with the hub region.^57,62,64^

## Results

### Demographic and Clinical Data

All demographic and clinical data are presented in Table 1 and in the Supplementary Results.

**Table 1.** Demographic and Clinical Data

	SCZ (*n* = 49)	BD (*n* = 49)	HC (*n* = 122)	Test-statistics
**Age**	36.53 ± 8.956	35.29 ± 9.028	31.26 ± 8.736	*F* = 7.95; *P* < .001
**Sex (female/male)**	12/37	21/28	59/63	Chi2 = 4.048; *P* = .019
**Race**				
** American Indian or Alaskan Native**	11 (22.4%)	4 (8.2%)	22 (18%)	
** Asian**	1 (2%)		2 (1.6%)	
** Black/African American**	2 (4.1%)	1 (2%)	1 (0.8%)	
** White**	32 (65.3%)	37 (75.5%)	96 (78.6%)	
** More than one race**	1 (2%)	7 (14.3%)		
**SANS**				
** Apathy-avolition**	2.69 ± 1.517	1.9 ± 1.388		*F* = 7.343; *P* = .008
** Anhedonia-asociality**	2.24 ± 1.493	1.69 ± 1.257		*F* = 3.946; *P* = .05
** Alogia**	0.94 ± 1.126	0.16 ± 0.426		*F* = 20.35; *P* *<* .001
** Blunted affect**	1.29 ± 1.307	0.57 ± 1.061		*F* = 8.824; *P* = .004
** Amotivation**	4.94 ± 2.764	3.54 ± 2.297		*F* = 7.314; *P* = .008
** Diminished expression**	2.22 ± 2.266	0.73 ± 1.366		*F* = 15.533; *P* < .001
**SAPS**				
** Positive symptoms**	4.92 ± 2.768	0.84 ± 1.419		*F* = 84.381; *P* < .001
** Disorganization**	2.59 ± 2.3	1.6 ± 1.765		*F* = 5.634; *P* = .02
**HAMD-21**	12.08 ± 9.02	13.8 ± 9.764		*F* = 0.815; *P* = .369
**Risperidone equivalent dose (mg/d)**	7.58 ± 8.855	4.9 ± 10.596		*F* = 1.843; *P* = .178

Abbreviations: BD, bipolar disorder I; HAMD-21, Hamilton Depression Scale (21 items); SANS, Scale for the Assessment of Negative Symptoms; SAPS, Scale for the Assessment of Positive Symptoms; SCZ, schizophrenia. SANS amotivation includes the SANS domains avolition-apathy and anhedonia/asociality. SANS diminished expression includes the SANS domains alogia and blunted affect. SAPS positive symptoms include the SAPS domains delusion and hallucination. SAPS disorganization includes the SAPS domains bizarre behavior and positive formal thought disorder.

## OFC Alterations and Amotivation across the Bipolar-Schizophrenia Spectrum

**Figure 2.**
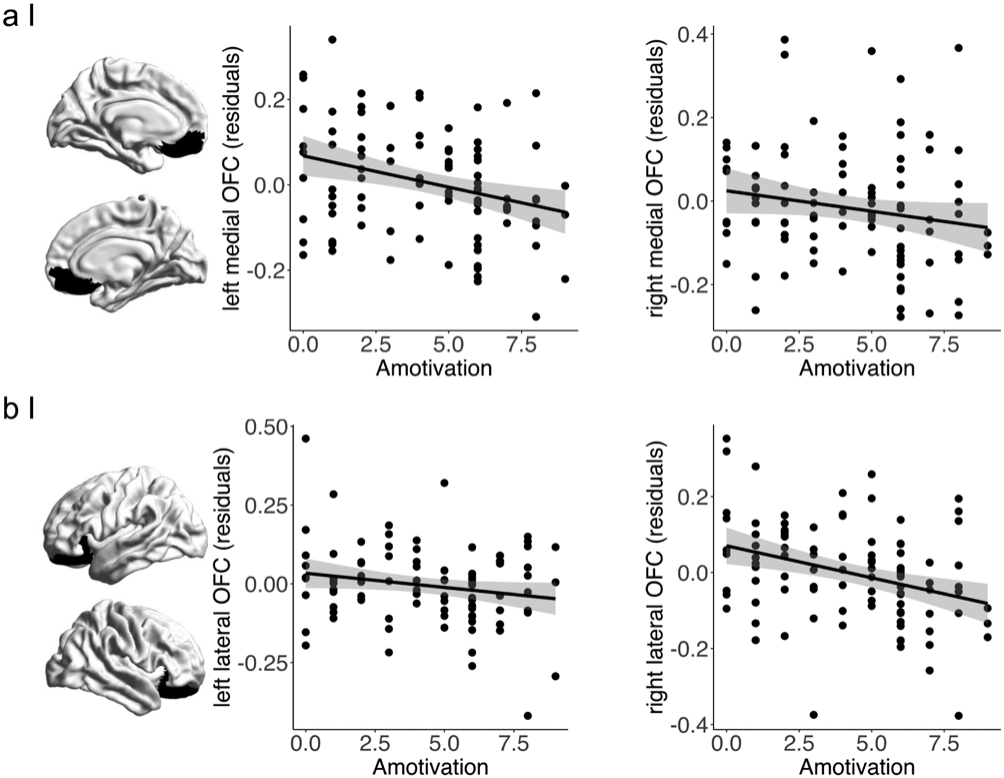
Transdiagnostic Correlation Analyses. (a) Associations between Medial OFC Thickness Residuals (Age and Sex Corrected) and Amotivation Severity. (b) Associations between Lateral OFC Thickness Residuals (Age and Sex Corrected) and Amotivation Severity. Error Shadings Correspond to Standard Errors. Significant Associations are Observed in Bilateral Medial OFC (Left: *rs *= −0.289, *p*_FDR_ = 0.008; Right: *rs *= −0.221, *p*_FDR_ = 0.039) and Right Lateral OFC (*rs *= −0.346, *p*_FDR_ = 0.002). Correlation Analysis between Amotivation and Left LOFC Residuals (Age and Sex Corrected) was Not Significant (*rs *= −0.121, *p*_FDR_ = 0.24).

Spearman correlations revealed significant negative correlations of amotivation with bilateral medial OFC thickness (left: *rs *= −0.289, *p*_FDR_ = 0.008; right: *rs *= −0.221, *p*_FDR_ = 0.039) and right lateral OFC thickness (*rs* = −0.346, *p*_FDR_ = 0.002) in both disorders combined (Figure 2). Reduced thickness of the left lateral OFC was not associated with higher amotivation across SCZ and BD (*rs* = −0.120, *p*_FDR_ = 0.24). Bootstrapping with 1000 resampled distributions further confirmed these associations with all 95% confidence intervals for the correlation coefficients not overlapping with zero (left medial [−0.47, −0.07]; right medial [−0.41, −0.01], and right lateral [−0.53, −0.15]). In contrast to the amotivation dimension, diminished expression was not significantly correlated with reduced OFC thickness but showed a trend negative association with reduced left lateral OFC thickness (Supplementary Table S7a).

To test whether associations with reduced OFC were specific for amotivation, we compared the correlation coefficients of amotivation and diminished expression with OFC thickness. We found that the correlation between right lateral OFC and amotivation was significantly different compared to the correlation between right lateral OFC and diminished expression (*Z *= −2.047, *P *= .021) but not for bilateral medial OFC (left: *Z *= −1.515, *P *= .065; right: *Z *= −1.249, *P *= .108). To test whether the transdiagnostic associations were comparable in both diagnostic groups, we repeated the correlation analyses in BD and SCZ separately. The associations between higher amotivation and reduced right lateral OFC as well as bilateral medial OFC thickness were confirmed in both groups separately (Supplementary Table S7b). Finally, exploratory correlation analyses with bilateral nucleus accumbens, caudate, and putamen volume did not reveal any significant associations with either negative symptom dimension (Supplementary Table S8). These null findings further emphasize that cortical, rather than subcortical, structural alterations may be more closely linked to amotivation. Altogether, we found that amotivation but not diminished expression was significantly associated with reduced right lateral OFC and bilateral medial OFC thickness across BD and SCZ.

## OFC Alterations and Other Clinical Factors

We further examined whether the association of the OFC was specific for amotivation or also related to other clinical factors, including risperidone equivalent dose, positive, disorganized, and depressive symptoms. Spearman correlations revealed significant negative correlations between risperidone equivalent dose and the reduced left lateral OFC (*rs *= −0.280, *P *= .005) as well as the right lateral OFC thickness (*rs *= -0.2, *P *= .046). To determine whether the observed association of amotivation and right lateral OFC thickness was confounded by antipsychotic medication, we performed partial correlation analysis including risperidone equivalent dose as a covariate. The correlation between amotivation and right lateral OFC thickness remained significant after including medication dose as a covariate (*rs *= −0.32, *p *= .002). None of the other clinical factors were significantly correlated with OFC thickness (see Supplementary Table S9). In summary, medication dose showed significant correlations with bilateral lateral but not medial OFC thickness; nevertheless, correlations between amotivation and right lateral OFC thickness remained significant after controlling for medication dose.

## OFC Covariance Networks and Negative Symptom Dimensions

**Figure 3.**
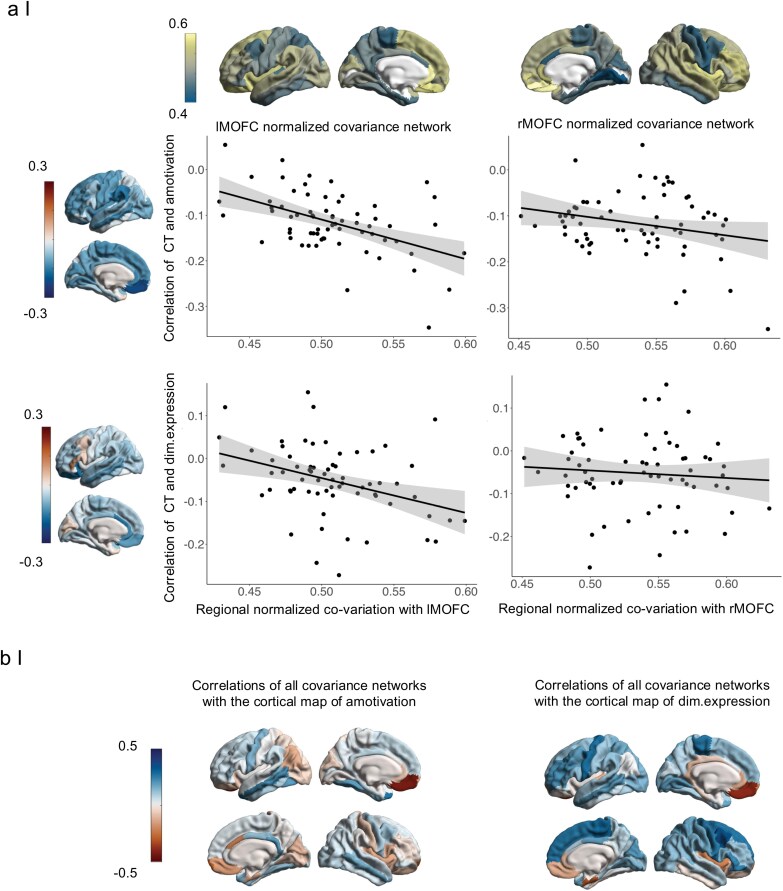
Association between Covariance Networks and Cortical Effect Sizes (Correlation *r*) of Amotivation across the BD-SCZ Spectrum. (a) Correlations between Left and Right Medial OFC Covariance Networks and Cortical Effect Size Maps of Amotivation and Diminished Expression, Respectively. Cortical Regions with Higher Degrees of Joint Variation with the Left Medial OFC Showed Higher Correlations with Amotivation Severity (*rs *= −0.43, *p*_spin_ = 0.001) and Diminished Expression Severity (*rs* = −0.38, *p*_spin_ = 0.004). Associations between the Right Medial OFC Covariance Network and the Cortical Maps of Amotivation (*rs* = −0.16, *p*_spin_ = 0.09) and Diminished Expression (*rs* = −0.13, *p*_spin_ = 0.195) Pointed in the Same Direction But were Not Significant. (b) Brain-wide Correlations between Each Regional Covariance Networks with the Spatial Pattern of Cortical Alterations Related to Amotivation and Diminished Expression, Respectively. Brain-wide Comparison Revealed that the Associations were Strongest for the Left Medial Covariance Network.

In the next step, we examined how OFC covariance networks relate to the brain-wide cortical alterations associated with amotivation and diminished expression across the BD-SCZ spectrum. The medial OFC covariance network showed that medial OFC thickness varies together with the thickness of proximal regions, including the ACC, insula, and other frontal and temporal areas, as well as some distant regions, such as the cuneus and lingual gyrus. In contrast, lower covariance with medial OFC thickness was found primarily in sensorimotor and parietal regions, as well as some more distant limbic areas connected to the OFC (Figure 3A). For visualization of the lateral OFC covariance networks see Supplementary Figure S5.

We found that the left medial OFC covariance network was significantly associated with the brain-wide cortical alterations (correlation strength) of amotivation (*rs *= −0.43, *p*_spin_ = 0.001). The association with the right medial OFC covariance network pointed in the same direction but was not significant (*rs* = −0.16, *p*_spin_ = 0.09) (Figure 3A). Bilateral lateral OFC covariance networks were not associated with the spatial pattern of cortical alterations related to amotivation (left/right *rs* = −0.08/−0.08, *p*_spin_ = 0.29/0.27) (Supplementary Figure S5). To assess whether associations with OFC covariance networks were symptom-specific for amotivation, we repeated the analysis with the diminished expression dimension and observed comparable relationships. The brain-wide cortical alterations associated with diminished expression (correlation strength) were significantly associated with the spatial pattern of the left medial OFC covariance network (*rs *= 0.38, *p*_spin_ = 0.004) but not with the right medial OFC covariance network (*rs *= −0.13, *p*_spin_ = 0.19) (Figure 3A). Bilateral lateral OFC networks were not associated with the spatial pattern of cortical alterations related to diminished expression severity (left/right *rs* = −0.003/−0.06, *p*_spin_ = 0.47/0.31; Supplementary Figure S5). We next assessed the correlation strengths of the left medial OFC covariance network compared to other regional covariance networks and systematically repeated the spatial correlation of the cortical alterations of amotivation with all other cortical covariance networks (*n* = 68 covariance networks) (Figure 3B). Ranking the correlation strengths, we found that the left medial OFC covariance network ranked first (Figure 3B; Supplementary Table S10). Brain-wide comparison of the association between left medial OFC covariance network and the spatial pattern of cortical alterations associated with diminished expression mirrored these findings (Figure 3B; Supplementary Table S11). Lastly, to show robustness of the observed association with OFC covariance networks, we repeated the analysis using independent data from 4474 individuals with schizophrenia from the ENIGMA consortium.^52^ In the ENIGMA schizophrenia sample (*n* = 4474), we generated independent OFC covariance networks and spatially correlated them with the meta-analytic brain-wide cortical alterations related to total negative symptoms. We found that bilateral lateral OFC covariance networks were significantly correlated with the spatial pattern of cortical alterations associated with total negative symptoms (left/right *rs* = −0.27/−0.25, *p*_spin_ = 0.04/0.05). No significant associations were observed for the medial OFC covariance networks (left/right *rs *= −0.08/0.05, *p*_spin_ = 0.31/0.37). While the ENIGMA findings did not confirm the medial OFC associations observed in the UCLA BD-SCZ sample, they suggest a broader spatial link between OFC covariance networks and cortical alterations related to negative symptom severity. Taken together, our findings indicate that OFC covariance networks are spatially associated with brain-wide cortical alterations of negative symptoms encompassing both the amotivation and diminished expression dimensions.

## Epicenter Mapping of Cortical Alteration Associated with Negative Symptom Dimensions

**Figure 4.**
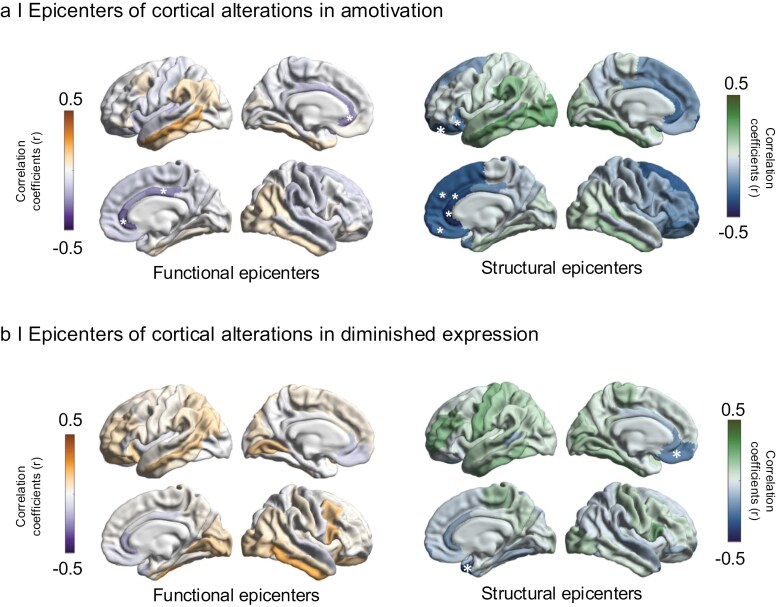
Epicenters of Brain-wide Cortical Alteration Patterns of Amotivation and Diminished Expression. Spatial Correlations between Brain-wide Cortical Alteration Associated with (a) Amotivation and (b) Diminished Expression and Seed-Based Cortico-cortical Connectivity of Each Region with All Other Regions were Used to Identify Epicenters of Symptom-Specific Cortical Alterations. Epicenters are Regions Whose Connectivity Profiles Significantly Spatially Correlated with the Cortical Alteration Map Related to Each Negative Symptom Dimension. We Assessed Statistical Significance Using Spin Permutation Tests (1000 Repetitions) and Repeated This Procedure Systematically to Assess the Epicenter Value of Every Cortical Region Using Both Functional and Structural Connectivity Matrices. (a) Correlation Coefficients Indexing Spatial Similarity between the Brain-wide Cortical Alterations of Amotivation and Seed-Based Functional (Left) and Structural (Right) Connectivity Measures for Every Cortical Region. The 5 Most Significant Epicenters are Highlighted with Asterisks. Functional Epicenters Included Bilateral rACC, Right PCC. Structural Epicenters Included Bilateral Frontal Pole Cortex, Right rACC, cACC, mOFC, and Superiorfrontal Cortex. (b) Correlation Coefficients Indexing Spatial Similarity between the Brain-wide Cortical Alterations of Diminished Expression and Seed-based Functional (Left) and Structural (Right) Connectivity Measures for Every Cortical Region. Significant Epicenters are Highlighted with Asterisks. No Significant Functional Epicenters were Identified. Structural Epicenters Included the Right Temporal Pole Cortex, and the Left mOFC.

We finally examined how the brain-wide cortical alterations associated with amotivation were spatially correlated with the normative functional and structural connectivity of the OFC and other cortical regions. With regards to cortico-cortical connectivity of the OFC, we found that structural connectivity of the right medial OFC (*r*_struc_ = −0.275, *p*_spin_ = 0.018) and left lateral OFC (*r*_struc_ = −0.251, *p*_spin_ = 0.003) was significantly correlated with the brain-wide cortical alterations associated with amotivation (Figure 4A; Supplementary Tables S12 and S13). Thus, cortical regions with strong connections to the right medial OFC and left lateral OFC showed relatively higher cortical thickness reduction in relation to amotivation severity. Beyond OFC connectivity, the right rostral anterior cingulate cortex (rACC) emerged as the strongest epicenter of the brain-wide cortical alterations of amotivation irrespectively of using functional or structural cortico-cortical connectivity (*r*_fun_ = −0.295, *p*_spin_ = 0.0098, *r*_struc_ = −0.410, *p*_spin_ = 0.001, Figure 4A; Supplementary Tables S12 and S13). In addition, significant functional and structural epicenters included other prefrontal and limbic areas adjacent to the OFC including bilateral frontal poles, the right caudal ACC, right PCC, and the right superior frontal gyrus (Figure 4A; Supplementary Tables S12 and S13). Exploring the spatial correlation between region-specific cortical-connectivity and the brain-wide cortical alterations related to diminished expression, we identified only the right temporal pole (*r*_struc_ = −0.348, *p*_spin_ = 0.005) and the left medial OFC (*r*_struc_ = −0.217, *p*_spin_ = 0.045) as putative epicenters (Figure 4B; Supplementary Tables S14 and S15). Taken together, the right medial and left lateral OFC as well as adjacent regions in particular the right rACC emerged as the most important epicenter of the brain-wide cortical alterations of amotivation. These epicenters were not significantly associated with the spatial pattern of cortical alterations associated with diminished expression. Overall, the brain-wide cortical alterations associated with amotivation were spatially more strongly anchored to region-specific cortico-cortical connectivity than the brain-wide cortical alterations associated with diminished expression.

## Discussion

Leveraging a transdiagnostic approach, we tested the hypotheses that OFC thickness alteration and OFC covariance networks are related to amotivation compared to diminished expression across the BD-SCZ spectrum. In addition, we assessed whether normative cortico-cortical connectivity contributes to the spatial pattern of brain-wide cortical alterations of both negative symptom dimensions. We found that reduced right lateral OFC and bilateral medial OFC thickness were specifically associated with amotivation but not with diminished expression. This relationship was independent of other clinical factors and was replicated within the BD and SCZ cohorts separately. Testing the association with cortical covariance networks, we observed negative associations between the left medial OFC covariance network and brain-wide alteration patterns related to amotivation and diminished expression. Repeating this analysis using independent meta-analytic data from the ENIGMA consortium revealed associations between bilateral lateral OFC covariance networks and total negative symptom severity. These findings indicate that the relationship between negative symptoms and cortical thickness reduction is not randomly distributed across the cortex but follows the structural covariance profiles of the OFC. Finally, we found that the cortico-cortical connectivity profiles of the right medial and left lateral OFC as well as adjacent regions including the right rACC were spatially associated with the cortical alteration related to amotivation severity. Collectively, this study identifies OFC alterations as a transdiagnostic morphometric signature of amotivation and offers insights into network mechanisms underlying the brain-wide cortical alteration pattern of negative symptoms across SCZ and BD.

Given the OFC’s pivotal role in decision-making processes,^87^ such as expected value and effort-cost computation, alterations in this area are widely recognized as important factors for motivational deficits in SCZ.^13,14,88^ Gold and colleagues initially demonstrated that impaired value representation is related to higher negative symptoms^26^ and more recently found that these impairments extend to maintaining reward-related information over time.^89^ Extending this work, several studies have shown that impaired value and effort-cost computation are associated with more severe negative symptoms^90^ and particular motivational deficits.^91–95^ Motivational deficits have been further linked to blunted OFC fMRI signals during reinforcement learning in SCZ^33^ and intertemporal decision-making across SCZ but also BD and major depressive disorders.^96^ Our data extend these behavioral and fMRI findings by providing a transdiagnostic link between neuroanatomical alterations of the OFC and motivational impairments across BD and SCZ. The observed relationships between lateral and medial OFC thickness reduction and amotivation scores are consistent with previous reports examining total negative symptoms in individuals with chronic SCZ^48^ and first-episode psychosis.^43^ Moreover, exploratory analyses revealed no significant associations between striatal subregion volumes and negative symptoms, further supporting the specificity of OFC alterations as a transdiagnostic morphometric signature of amotivation. By deconstructing different negative symptoms, our findings suggest a stronger association between OFC thickness reduction and amotivation compared to the diminished expression dimension. It should be noted, however, that previous work has reported comparable associations of OFC alterations with both negative symptom dimensions in first-episode psychosis.^43^ Rather than being mutually exclusive, these results may provide a complementary picture of the involvement of the OFC in the pathogenesis of amotivation and diminished expression. The OFC is embedded in multiple cortico-cortical and cortico-subcortical networks and participates in a plethora of complex functions, including the processing of emotions.^87,97–99^ Alterations in the OFC may therefore represent both a key substrate of motivational deficits within the BD-SCZ spectrum, as well as play a role in the development of diminished emotional expressivity. However, contrary to the accumulating evidence supporting compromised OFC contributions to decision-making and reward learning in association with motivational deficits, studies linking impaired OFC signaling during emotional processing within the SCZ spectrum are relatively scarce.^13,14,88^ Thus, future research should combine decision-making and emotion-processing tasks with multimodal neuroimaging to disentangle how distinct OFC abnormalities contribute to both motivational deficits and diminished expressivity across the psychosis continuum.

We further identified that the brain-wide cortical alterations associated with amotivation were not randomly distributed but constrained within the covariance network of the left medial OFC. This association was not specific for amotivation but was also observed with the brain-wide cortical alterations related to diminished expression suggesting a general mechanism for both negative symptom dimensions. Complementary analysis using independent meta-analytic data from the ENIGMA consortium extended these findings by showing significant associations of lateral OFC covariance networks with the cortical alteration pattern associated with total negative symptoms. While these findings do not confirm the left medial OFC covariance network associations observed in the UCLA BD-SCZ sample, they align with the relationships of medial and lateral OFC thickness reduction and negative symptoms observed in the present and previous work.^36,48^ Importantly, rather than a specific OFC subregion being the key driver of these spatial associations, our findings across both analyses suggest that multiple OFC subregions may be involved in shaping the cortical alteration patterns related to negative symptoms. Future studies using a more granular, regionally refined approach to OFC covariance networks may help better characterize a potential gradient of involvement along the OFC, providing further insight into its role for both negative symptom dimensions. In addition, the regional variations in OFC covariance network associations may be influenced by methodological and sample-dependent differences between the transdiagnostic UCLA BD-SCZ sample and the meta-analytic ENIGMA SCZ dataset. Key differences include the assessment of negative symptoms, amotivation and diminished expression were examined separately in the UCLA BD-SCZ sample, whereas total negative symptom severity was used in the ENIGMA meta-analysis. In addition, the meta-analytic data from the ENIGMA study only include individuals with SCZ.

Cortical covariance networks reflect coordinated structural changes across brain regions and are shaped by maturational coupling and trophic processes during cortical development.^82,84,100,101^ Covariance networks have been shown to describe coordinated patterns of cortical atrophy in neuropsychiatric disorders, including schizophrenia,^54,84,102^ and different networks may relate to distinct symptom dimensions.^55,56^ In line with these insights, our findings suggest that OFC covariance networks contribute to the brain-wide cortical alterations associated with negative symptoms, though the specific associations appear to depend on methodological and sample characteristics. One possible explanation is that genetic factors and trophic processes shaping medial and lateral OFC covariance networks contribute to the pathophysiology of negative symptom-related cortical alterations through their link with reward-based functional networks. Investigating the cellular and molecular mechanisms shaping these structural networks may help identify disrupted biological systems underlying negative symptoms in the BD-SCZ spectrum.^84^

We finally contextualized the contribution of normative brain network architecture to the spatial pattern of cortical alterations associated with amotivation in BD and SCZ. A growing body of literature provides evidence that cross-disorder and disease-specific cortical alteration patterns of neuropsychiatric disorders are guided by normative connectivity profiles of distinct cortical regions.^63^ In schizophrenia, temporo-paralimbic regions have been identified as putative epicenters whose connectivity profile spatially constrains the extent of cortical atrophy.^57,58,63^ Analysis across different disease stages further suggests that epicenters of cortical atrophy emerged first in occipital regions early in the illness and shifted to prefrontal regions with illness progression.^57,63^ Cross-disorder comparison of BD and SCZ revealed convergence of parieto-temporal and frontal epicenters of cortical alterations seen in both disorders. Furthermore, higher psychotic symptom severity has been shown to be associated with sensory-motor and paralimbic epicenters suggesting that individual clinical facets contribute to the development of distinct symptom-specific epicenters.^57^ Extending these observations, our findings indicate that the brain-wide cortical alterations associated with amotivation in BD and SCZ are anchored to the connectivity profiles of prefrontal and limbic areas. The right medial OFC, left lateral OFC, and in particular the right rACC emerged as significant epicenters of the cortical alteration pattern associated with amotivation. The rACC is tightly connected to proximal regions, in particular the OFC, and other areas of the ACC and prefrontal cortex.^103^ In addition, it is a major hub of large-scale networks such as the default mode network.^104^ This position at the intersection between motivation and cognitive control networks aligns with the important function of the rACC in value-based decision-making.^105–107^ Altered structure and function of the ACC has been consistently reported to be associated with motivational deficits across neuropsychiatric disorders including schizophrenia and affective disorders.^13,15,37^ Our findings demonstrate that the spatial pattern of cortical alterations associated with amotivation is not randomly distributed but determined by the functional and structural connectivity profile of the OFC and ACC. This is consistent with the growing recognition that pathological processes in psychiatric disorders follow network principles and are related to the underlying connectome architecture.^53,108,109^ Although it is not possible to deduce such mechanisms from the present findings, one hypothesis could be that the OFC and ACC act as gateways from which altered neurodevelopment or neurodegeneration propagates to the connected areas.

## Limitations and Future Directions

Capitalizing on BD-SCZ spectrum data, we provide support for transdiagnostic orbitofrontal morphometric signatures of amotivation and network characteristics linked to the brain-wide cortical alterations associated with negative symptoms. The sample size might have limited the possibility to capture more subtle associations, particularly in disentangling regional and gradual effects of the network-level alterations. While our study focused on negative symptoms, studying other symptom dimensions such as manic symptoms would have provided additional insights into the broader clinical phenotype in BD. Therefore, larger BD-SZ spectrum samples including different stages of both conditions are needed (1) to replicate our findings, (2) to study the role of localized and network alterations of the OFC and ACC during early disease courses, and (3) to extend our approach to other shared symptom dimensions across the BD-SC spectrum. Although a harmonized assessment of negative symptoms was used by applying the SANS to both diagnostic groups, a multimodal approach including newer scales, self-reports, and behavioral measures would be valuable for future research. The severity of the amotivation dimension and diminished expression were comparable to previous psychometric and neuroimaging studies in the BD-SCZ spectrum,^22–25,43^ therefore, allowing for comparison with the existing literature. However, defining a priori thresholds of negative symptom severity might help to better examine brain-behavior relationships across the full range of negative symptom severity. In addition, the complementary analysis using meta-analytic ENIGMA data had inherent limitations, including methodological differences in negative symptom assessment and the exclusive inclusion of SCZ cohorts, which may have influenced the observed covariance network associations. Finally, if replicated, our findings could have important translational implications for treatment. The identified transdiagnostic network alterations associated with amotivation may inform patient stratification in transdiagnostic clinical trials on negative symptoms, helping to identify individuals who might benefit from targeted interventions.^110^ In addition, the observed orbitofrontal and network-level alterations could be explored as potential neuroanatomical targets for neuromodulation approaches such as transcranial magnetic stimulation (TMS),^111^ guiding personalized treatment strategies for negative symptoms across the BD-SCZ spectrum.

## Conclusion

Leveraging a combined BD-SCZ sample, we found that reduced OFC thickness reflects a transdiagnostic signature of amotivation severity. Notably, these associations were not related to other symptoms or specific to one diagnostic group, highlighting its potential utility as a target for transdiagnostic patient stratification and treatment development. Our results further suggest a link between distinct OFC covariance networks and connectivity profiles with brain-wide cortical alteration patterns of amotivation and diminished expression. More broadly, our work contributes to the growing recognition that neuropsychiatric psychopathologies can be characterized by alterations in circumscribed cortical networks that are linked to distinct network features of the brain’s connectome architecture.

## Supplementary material

Supplementary material is available at https://academic.oup.com/schizophreniabulletin

sbaf078_suppl_Supplementary_Tables_1-15_Figures_1-5

## Data Availability

All data used in this study were derived from the UCLA CNP cohort. Data can be downloaded from the publicly available database OpenfMRI (https://openfmri.org/dataset/ds000030/).
