# Supplementary material for: Orbitofrontal Thickness and Network Associations as Transdiagnostic Signature of Amotivation Along the Bipolar-Schizophrenia Spectrum
Source: Schizophr Bull. 2025 Jun 20;52(2):sbaf078. doi: 10.1093/schbul/sbaf078 (PMC12996912; doi:10.1093/schbul/sbaf078)
Supplement: sbaf078_suppl_Supplementary_Tables_1-15_Figures_1-5 [file sbaf078_suppl_supplementary_tables_1-15_figures_1-5.pdf]

Altered OFC structure and interregional covariance as transdiagnostic signature of amotivation along the bipolar-schizophrenia spectrum

Supplement

Methods

Image Acquisition and Processing

Imaging data were collected with one of two 3T Siemens Trio scanners. One was located at the Ahmanson-Lovelace Brain Mapping Center (Siemens version syngo MR B15) and the other one at the Staglin Center for Cognitive Neuroscience (Siemens version syngo MR B17) at UCLA. High-resolution anatomical scans were collected with a T1-weighted matched-bandwidth sequence with the following parameters: 4 mm slices, TR/TE = 5000/34 ms, 4 averages, matrix = 128×128. 90 degree flip angle. We used FreeSurfer Version 5.3.0 recon-all function to perform cortical reconstruction and volumetric segmentation. The automated recon-all function processing streamline for structural MRI data includes motion correction, removal of non-brain tissue, automated Talairach transformation, segmentation of the subcortical white matter and deep gray matter volumetric structures, intensity normalization, tessellation of the gray matter-white matter boundary, topology correction, and surface generation and deformation. Quality Control (QC) was performed using standard ENIGMA QC protocols (http://enigma.ini.usc.edu/protocols/imaging-protocols).

Group Comparison

Group comparisons of standardized residuals of OFC thickness from HC, BD, SCZ (total n = 220) were performed using one-way univariate analysis of covariance (ANCOVA). T-tests were used for subsequent post hoc analyses (IBM SPSS Version 28.0.1.1 (14). Results can be found in Table S2.

Results

Demographic and clinical data

All demographic and clinical data are presented in Table 1. There was no group difference in age [*F(1, 96)* = 0.47, *p* = 0.495], but trend-level higher proportion of male subjects in the SCZ (75%) group compared to the BD group (57%, chi2 = 3.701, p = 0.054). In both SCZ and BD, levels of negative symptoms were mild to moderate, with higher amotivation [*F(1, 95)* = 7.31, *p* = 0.008] and diminished expression severity [*F(1, 96)* = 15.53, *p* <0.001] in SCZ compared to BD. With respect to the other symptom dimensions, we found higher SAPS positive symptom dimension scores [*F(1,96)* = 84.381, *p* < 0.001] as well as higher levels in SAPS disorganization [*F(1,94)* = 5.634; *p* = 0.02] in the SCZ group than the BD group. In contrast, HAMD-21 scores did not differ between groups [*F(1,96)* = 0.815; *p* = 0.369]. Regarding antipsychotic medication dose, we found no significant difference between the SCZ and the BD group [*F(1,96)* = 1.843; *p* = 0.178].

Table S1. Mean values of cortical thickness across all datasets

Region	HC (n = 122)	BD (n = 49)	SCZ (n = 49)
L LOFC	2.49 (0.12)	2.48 (0.14)	2.46 (0.134)
R LOFC	2.42 (0.122)	2.4 (0.145)	2.38 (0.14)
L MOFC	2.3 (0.123)	2.29 (0.143)	2.30 (0.131)
R MOFC	2.24 (0.112)	2.16 (0.146)	2.19 (0.162)

Table S1: Data are presented as means and standard deviations of cortical thickness (mm). HC, healthy controls; BD, patients with bipolar disorder; SZ, patients with schizophrenia; LOFC, lateral orbitofrontal cortex; MOFC, medial orbitofrontal cortex; L, left; R, right

Table S2. Group differences in standardized residuals of OFC thickness

Region	HC (n = 122)	BD (n = 49)	SCZ (n= 49)	df	Error	F	p-value	Post-hoc test[a]	Post-hoc p-value[a]
L LOFC	0.0032	0.009	-0.0165	2	217	0.656	0.520		
R LOFC	0.001	0.006	-0.009	2	217	0.205	0.815		
L MOFC	-0.004	-0.004	0.014	2	217	0.399	0.672		
R MOFC	0.014	-0.039	0.003	2	217	3.19	**0.043**	HC > BD<br>HC=SCZ<br>BD=SCZ	**0.038**<br>0.593<br>0.197

Table S2. Data are presented as standardized residuals, age and sex regressed-out for orbitofrontal cortical thickness. Standard error in parenthesis. Univariate analyses were corrected for site. Mean values of cortical thickness (mm) are presented in Table 2. HC, healthy controls; BD, patients with bipolar disorder; SZ, patients with schizophrenia; LOFC, lateral orbitofrontal cortex; MOFC, medial orbitofrontal cortex; L, left; R, right; Significant group differences in bold. [a]Post-hoc tests are corrected for multiple comparisons using the Holm-Bonferroni method (FWE p<0.05).

Table S3. ENIGMA Sample demographics adapted from van Erp et al.[1]

Sample	N (Total)	N (SZ)	N (HV)	M/F (SZ)	M/F (HV)	Mean Age (SZ)	Mean Age (HV)	Mean Prenatal SES (SZ)	Mean Prenatal SES (HV)	Mean Age of Onset	Mean Duration Of Illness	PANSS Total	PANSS Negative	PANSS Positive	SANS Total	SAPS Total
AMC	292	196	96	170/26	63/33	22.9	22.2			20	2.2					
ASRB	429	263	166	177/86	79/87	39.3	38.6			23.6	14.6				18.2	
CAMH	264	118	146	70/48	77/69	43.6	43.9	40	47.7	24.9	19.2	53.1	14	13.9		
CIAM	51	21	30	13/8	16/14	26.6	31			23.2	7.4	54.6	14.8	13.6		
CLING	372	49	323	36/13	132/191	25.2	32.4	14.9	17.7	24.5	7.7	49.8	12	11.3		
COBRE	142	72	70	59/13	50/20	35.7	37.2			21.5	15.5	60	14.9	15.2		
Dublin	277	55	222	39/16	95/127	29.2	42.6			23	20.2				21.6	19.4
ESO	80	40	40	20/20	20/20	29.1	29.4			28.9	0.7	63.5	16.1	14		
EdinburghEHRS	67	31	36	19/12	17/19	21.2	21.8	2.8	3.4							
EdinburghFunc	60	25	35	11/14	18/17	37.5	37.2	0.6	0.1	22.2	15.5	43	10	10.6		
EdinburghSFMH	76	35	41	23/12	23/18	38.2	37.5	3.9	4	23	14.4	56.4	14.1	13.4	27.9	
FBIRN	359	185	174	139/46	124/50	37.5	38.9	5.2	5.6	21.8	17.2	58.6	14.5	15.5	19.6	16.7
FIDMAG	283	160	123	124/36	54/69	37.5	39.6			23.3	15.4	76.5	22.9	16.7		
Frankfurt	59	29	30	20/9	13/17	35.2	38.1	13.7	14.7	35.6	10.9	67.2	16.7	16.9		
GAP	212	124	88	87/37	33/55	25.9	27.4					62.3	16.4	15.7		
Galway	129	66	63	49/17	41/22	33.7	32.9			23.5	9.7				27.4	14.8
HMS	101	46	55	32/14	28/27	35.4	28.4	12.9	13			90.2	22.1	21.1		
HUBIN	196	94	102	70/24	69/33	42	41.7			24.5	17.1				22.3	9
Huilong1	190	154	36	83/71	19/17	31.8	26.2									
Huilong2	140	90	50	47/43	28/22	24.8	24.3									

KaSP	88	56	32	34/22	15/17	27.5	30.3				1.2	74	17.1	18.6		
MCIC	312	148	164	113/35	102/62	31.4	32.9	2.8	2.7	22.8	10.2				23.3	22.8
MPRC1	227	82	145	47/35	57/88	34.7	33.6									
MPRC2	165	88	77	44/44	28/49	41.7	37.7									
NU	200	108	92	74/34	51/41	31.9	34.2	14.1	14.5	20.8	13.2				33	23.2
OLIN	870	313	557	174/139	311/246	37.6	37.7									
Osaka	824	204	620	109/95	315/305	34.3	35.5	4.9	5.1	24.3	11.3	81.2	19.8	18.7		
PAFIP1.5T	222	142	80	88/54	50/30	27.7	29.7	3.7	3.5	28.5	1				6.4	13.6
PAFIP3T	218	114	104	64/50	63/41	30.1	29.7	3.4	3.9	28.9	0.7				5.5	14.1
RSCZ	94	45	49	45/0	49/0	22.2	22.1	NaN	NaN	21.2	1.2	60.9	19.3	11.2		
RomeSL	289	173	116	118/55	73/43	37.5	39.2	NaN	NaN	24.3	15	87.8	21	21.1	31.7	32.3
SCORE	205	161	44	117/44	17/27	25.5	25.5	NaN	NaN	24.5	1.1				15.6	
SNUH	139	43	96	20/23	58/38	25.8	22.5	2.7	2.8	22.2	0.6	68.5	17.4	16.5		
SaoPaulo	84	15	69	9/6	45/24	27.3	24.1	NaN	NaN			89.9	21.9	22.6		
TOP	522	219	303	130/89	159/144	35.4	32	NaN	NaN	23.9	8.3	62	15.5	14.9		
UMCU	609	322	287	241/81	166/121	32.9	30.8	12.4	12.6	21.8	9	65.4	16.4	15.9		
UMCUS	190	123	67	72/51	27/40	39.7	37.2	10.7	12.7			66.6	16.7	17.4		
UNIBA	165	88	77	65/23	31/46	26.6	33.6	27.2	40.9	20.5	12.4	77.8	21.7	17.3		
UPENN	370	177	193	105/72	90/103	36.4	38.9	13.3	13.8	20.9	17.4				23.8	18.3
Total	9572	4474	5098	2957/1517	2706/2392			NaN	NaN							
Sample size-weighted mean						32.3	34.5	NaN	NaN	23.4	10.5	68.1	21.9	16.4	20.5	19.2

N=Number; SZ=Schizophrenia; HV=Healthy Volunteer; M=Male; F=Female; SES=Socioeconomic Status; PANSS=Positive and Negative Syndrome Scale; SANS=Schedule for the Assessment of Negative Symptoms; SAPS=Schedule for the Assessment of Positive Symptoms

Table S4. ENIGMA Sample medication information adapted from van Erp et al.[1]

Sample	N Unmedicated	N Second Generation (atypical)	N First Generation (typical)	N Both (typical + atypical)	Mean CPZ
AMC	26	151	18	0	250.1
ASRB	44	198	12	9	
CAMH	19	84	7	8	288.6
CIAM	4	9	5	3	
CLING	9	35	0	5	637.8
COBRE	0	61	7	1	548.2
Dublin	0	25	1	5	358.3
ESO	3	11	9	6	
EdinburghEHRS	0	15	8	2	
EdinburghFunc	11	23	1	0	590.3
EdinburghSFMH	0	29	0	2	309.9
FBIRN	0	137	20	10	373.3
FIDMAG	1	100	7	30	634.6
Frankfurt	0	26	3	0	605.1
GAP	2	36	1	0	198.8
Galway	0	12	0	3	469.7
HMS	6	39	0	1	313
HUBIN	6	38	40	10	272.7
Huilong1	0	54	0	11	
Huilong2	0	37	0	0	
KaSP	29	25	0	2	

MCIC	8	117	10	7	533.5
MPRC1	-	-	-	-	
MPRC2	-	-	-	-	
NU	9	79	17	0	
OLIN	-	-	-	-	
Osaka	18	132	8	46	642.6
PAFIP1.5T	0	0	117	25	199.1
PAFIP3T	0	114	0	0	166.5
RSCZ	17	88	29	39	
RomeSL	0	0	0	0	385
SCORE	15	0	0	0	203.2
SNUH	121	38	2	0	
SaoPaulo	10	32	0	1	
TOP	28	158	5	18	404.4
UMCU	28	171	87	5	
UMCUS	11	50	16	2	
UNIBA	0	47	4	9	639.1
UPENN	0	65	13	5	481.9
Total	425	2236	447	265	
Sample size-weighted mean					399

Table S5. Sample Image Acquisition and Image Processing Details adapted from van Erp et al.[1]

Sample	Number Of Scanners	Scanner Vendor & Type	Imaging Protocols	Slice Orientation	FreeSurfer Version	Operating System	Number of subjects removed from analysis due to QC failure?
**AMC**	1	Philips Intera 3T	TR: 8-9.8, M=9,4 (0,41). TE:3,5-4,6, M=4,26 (0,46). Slice thickness: 1/1.2, flip angle: 8degr, rows/columns: 192-288, M=255,49 (7,00). Pixel spacing: 1mm		v5.0.0	Linux centos4 x86_64	1
**ASRB**	5	Siemens Avanto 1.5T	High-resolution T1-weighted structural magnetic resonance imaging (sMRI) brain scans (MPRAGE) were acquired using an optimized magnetization-prepared rapid acquisition gradient echo on 1.5 T Siemens Avanto scanners (Siemens, Erlangen, Germany) across five Australian research sites (Loughland and al., 2010). Image parameters were set to 176 slices of 1mm thickness, no gap with field-of-view 250 x 250 mm^2, repetition time 1980 ms, echo time 4.3 ms, data acquisition matrix 256 x 256, with a flip matrix of 15°, resulting in a voxel size of 0.98×0.98×1.0 mm^3	Sagittal	v5.1.0	Mac OSX	0

**CAMH**	1	GE 1.5T	SPGR, TR/TE/TI=12.3/5.3/300ms, flip angle=20°, 256x256x128 matrix, FOV=240x240mm, slice thickness=1.5mm	Axial	v5.3.0	xubuntu x86_64-linux	0
**CIAM**	1	3T Siemens Allegra	MPRAGE (van der Kouwe et al., 2008) sequence :TR = 2530 ms, graded TE = 1.53, 3.21, 4.89, 6.57 ms, flip angle = 7°, FOV = 256 mm, slice thickness = 1.33 mm, 128 slices, voxel size 1.3x1.0x1.3, scan time 8:06. Single channel coil used	Sagittal	v5.3.0	Linux	3
**CLiNG**	1	3T Magnetom TIM Trio	MRI scanning was performed on a 3.0-Tesla Magnetom TIM Trio (Siemens, Erlangen, Germany). A T1-weighted, 3D magnetization prepared rapid gradient echo sequence (MPRAGE) (TR/TE/TI/FA=2250 ms/3.26 ms/900 ms/9°; image matrix = 256 x 256; duration 8 min and 26 sec) was acquired generating 192 sagittal slices with a voxel size of 1 mm3.”	Sagittal	v5.3.0	Ubuntu 12.04	0
**COBRE**	1	3T Siemens TIM Trio	T1-weighted images were acquired with a 5-echo multi-echo MPRAGE sequence [TE (echo times) = 1.64, 3.5, 5.36, 7.22, 9.08 ms, TR (repetition time) = 2.53 s, TI (inversion time) = 1.2 s, 7○ flip angle, number of excitations (NEX) = 1, slice thickness = 1 mm, FOV (field of view) = 256 mm, resolution = 256x256]	Sagittal	v5.3.0	Linux RedHat	

**Dublin**	1	3T Philips Intera Achieva	180 slice T1-weighted image using a TFE gradient echo pulse sequence (TR=8.4ms, TE=3.8ms, flip angle=8°, slice thickness=0.9mm, voxel size=0.9mm^3, 180slices, duration=6min)	Axial	v5.3.0	Linux	0
**ESO**	1	3T Siemens Tim Trio	MP-RAGE 3D, 1mm thickness, acquisition matrix 256 x 256, TR=2300ms, TE=4.63ms,TI=900ms	Sagittal	v5.3.0	Linux	Only subjects without significant motion artifacts (assessed by visual inspection) were included. Apart from ENIGMA QA protocol, visual inspection of all slices and edits to the skullstrip, white matter segmentation and control points insertion for correction of signal intensity normalization were done where needed.
**EdinburghEHRS**	1	1T Siemens	scanned with a 1 Tesla 42 SPE Siemens MRI scanner (Siemens, Erlangen,Germany). 128 contiguous coronal T1-weighted slices (thickness1.88 mm,field-of-view 250 × 250 mm) were obtained using aMagnetisation Prepared Rapid Acquisition of Gradient Echo (MPRAGE)sequence (TR=10ms, TE=4ms, TI=200ms, relaxation time 500ms).	Coronal	v5.3.0	Linux	0 Removed. All scans were manually checked for inaccuracies by a trained rater blinded to diagnostic status. At this stage, editing procedures outlined on the freesurfer wiki (http://freesurfer.net/fswiki/Edits) were then performed on all scans to remove non-brain from brain, and white matter edits to increase the accuracy of the pial surface. Control points were added when normalization steps failed.
**EdinburghFunc**	1	1.5T GE Signa	A coronal gradient echo sequence with magnetization preparation and produced 128 coronal high-resolution T1-weighted images, which were used for structural image analysis (time of inversion [TI]	Axial	v5.1.0	Linux	0

			600 msec, echo time 3.4 msec, flip angle 15, field of view 22, slice thickness 1.7 mm, matrix 256 192).				
**EdinburghSFMH**	1	3T Siemens Verio	Used T1-weighted, magnetisation prepared rapid acquisition gradient echo (MP-RAGE) sequence prescribed using the AC-PC line, providing 160 saggital slices of 1.0mm thickness, with 256 x 256mm2 field of view, matrix size 256 x 256mm2. Further scan parameters – repetition time = 2300ms, echo time = 2.98ms, inversion time = 900ms and flip angle = 9degress.	Sagittal	v5.3.0	Linux	0
**FBIRN (Phase3)**	7	3T Siemens Tim Trio; 3T GE	High-resolution structural imaging scans were acquired on six 3T Siemens Tim® Trio System and one 3T General Electric Discovery MR750 scanner. MP-RAGE scan parameters for the Siemens scanner were: scan plane=sagittal, TR/TE/TI=2300/2.94/1100ms, GRAPPA acceleration factor=2, flip angle=9°, resolution=256×256x160, FOV=220mm2, voxel size=0.86x0.86x1.2mm, and NEX=1. IR-SPGR scan parameters for the General Electric scanner were: scan plane=sagittal, TR/TE/TI=5.95/1.99/450ms, ASSET acceleration factor=2,	Sagittal	v5.3.0	Centos 64bit	0

			a flip angle=12°, resolution=256×256x166, FOV=220mm2, voxel size=0.86x0.86x1.2mm, and NEX=1. All scans covered the entire brain.				
**FIDMAG**	1	1.5T GE Signa	180 axial slices; 1mm slice thickness, no gap, matrix size 512x512; 0.5x0.5x1mm3 voxel resolution; TE 4ms, TR 2000ms, flip angle 15º	Axial	v5.3.0	Linux Ubuntu	
**Frankfurt**	1	3T Siemens Trio	176 slices, slice thickness 1mm, TR= 7.92 ms, TE= 2.48 ms, voxel resolution= 1x1x1 mm, flip angle= 16°	Sagittal	v5.1.0	Linuxaxia	
**GAP**	1	3T GE Signa HDx	SAGITTAL ADNI MPRAGE GE, slice thickness = 1.2mm, spatial positions = 166 slices, flip angle = 8°, fov = 260mm x 260mm, TR/TE/TI = 6.988/2.848/650ms, matrix = 256mm x 256mm		v5.3.0		3 subjects were excluded because of motion artifcacts, 7 subjects were excluded after QC, Pial edits conducted to remove small parts of non-brain matter that were included in the brainmask in 37 subjects.
**Galway**	1	1.5 Tesla Siemens Magnetom Symphony (Erlangen. Germany)	A volumetric T1-weighted magnetization-prepared rapid acquisition of gradient echo (MPRAGE) sequence was acquired with the imaging parameters: Repetition time (TR): 1140ms, Echo time (TE): 4.38ms, flip angle 15; matrix size 256 x 256; an in-plane pixel resolution of 0.9mm x 0.9mm and slice thickness 0.9mm	Axial	v5.1.0	Linux	0

**HMS**	1	1.5 T Magnetom Sonata	MRI scanning was performed on a 1.5-Tesla Magnetom Sonata (Siemens, Erlangen, Germany). A T1-weighted, magnetization prepared rapid gradient echo sequence (MPRAGE) (TR/TE/TI/FA=1900 ms/4.0 ms/700 ms/15°; image matrix = 256 x 256) was acquired generating 176 consecutive sagittal slices with a voxel size of 1 mm3. ~5 min	Sagittal	v 5.1.0	centos6 x86_64	0
**HUBIN**	1	1.5 Tesla General Electronics Signa	T1-weighted images, using a three-dimensional spoiled gradient recalled (SPGR) pulse sequence, were acquired with the following parameters; 1.5 mm coronal slices, no gap, 35° flip angle, repetition time (TR) = 24 ms, echo time (TE) = 6.0 ms, number of excitations (NEX) = 2, field of view (FOV) = 24 cm, acquisition matrix = 256 × 192. T2-weighted images were acquired with the following parameters; 2.0 mm coronal slices, no gap, TR = 6,000 ms, TE = 84 ms, NEX = 2, FOV = 24 cm, acquisition matrix = 256 × 192.	Coronal	v5.3.0	RedHat	0
**Huilong1**	1	3T Siemens Verio	T1-weighted, 3D MPRAGE, 1x1x1mm, TE/TR/TI=.9/2300/900ms, flip angle=9 degrees.	Sagittal	v5.3.0	Linux	

**Huilong2**	1	3T GE Signa HDxt	T1-weighted, 3D BRAVO, 1x1x1mm, TE/TR/TI=2.5/6.8/1100ms, flip angle=7 degrees.	Sagittal	v5.3.0	Linux	
**KaSP**	1	3T GE	3D IR prep fast SPGR, TR=7.904ms, TE=3.06ms, TI = 450ms, flip angle = 12, 146 slices, voxel size = 0.934 x 0.934 x 1.2 mm3, matrix = 256 x 256	Sagittal	v5.3.0	Linux Hat Etnerprise 6.5	
**MCIC**	3	1.5, 3T Siemens and GE	T1 scans:<br>TR = 2530 ms for 3 T, TR = 12 ms for 1.5 T; TE = 3.79 ms for 3 T, TE = 4.76 ms for 1.5 T; FA = 7 for 3 T, FA = 20 for 1.5 T; TI = 1100 for 3 T; Bandwidth = 181 for 3 T, Bandwidth = 110 for 1.5 T; 0.625×0.625 mm voxel size; slice thickness 1.5 mm; FOV 256×256×128 cm matrix;<br>FOV = 16 cm (could be increased to 18 cm when needed<br>for full brain coverage).	Coronal	v4.0.1	Linux of various flavors	5 subjects failed automated segmentation procedure due to excessive motion artifacts<br>2 participants' MRI data failed the manual inspection
**MPRC1**	1	3T Siemens Allegro	T1-weighted, 3D MPRAGE, 1x1x1mm, TE/TR/TI=4.3/2500/1000ms, flip angle=8 degrees.	Sagittal	v5.3.0	Linux	
**MPRC2**	1	3T Siemens Trio	T1-weighted, 3D MPRAGE, 1x1x1mm, TE/TR/TI=2.9/2300/900ms, flip angle=9 degrees.	Sagittal	v5.3.0	Linux	
**NU**	1	1.5T Vision	1) 3D turbo-FLASH: TR=20 ms, TE=5.4 ms, flip=30˚, ACQ=1, 256x256 matrix, 1xl	Axial	v5.3.0	centos6 x86_64	

			mm in-plane resolution, 180 slices, slice thickness 1 mm, 13:30 min scan time and 2) 3D MPRAGE (2-4 repeats): TR=9.7 ms, TE=4 ms, flip=10˚, ACQ=1, 256x256 matrix, 1xl mm in-plane resolution, 128 slices, slice thickness 1.25 mm, 5:36 min scan time each				
**OLIN**	1	3T Alegra	T1-weighted, 3D magnetization-prepared rapid gradient-echo (MPRAGE) sequence (TR/TE/TI=2200/4.13/766 ms, flip angle=13°, voxel size [isotropic]=0.8mm, image size=240 x 320 x 208 voxels), with axial slices parallel to the AC-PC line.	Axial	v5.1.0		
**Osaka**	2	1.5T GE Signa Excite, 3T GE Signa HDxt	3D-IR-FSPGR, TR/TE/TI=12.6/4.2/400ms, flip angle=15°, 256x256x124 matrix, FOV=240x240mm, slice thickness=1.4mm, Nex=1, No Asset, QD Head coil; 3D-IR-FSPGR, TR/TE/TI=7.2/2.9/400ms, flip angle=11°, 256x256x172 matrix, FOV=240x240mm, slice thickness=1.0mm, Nex=1, No Asset, 8ch Brain coil	Sagittal	v5.3.0	SUSE Linux Enterprise Server 10; Red Hat Enterprise Linux 6	
**PAFIP1.5T**	1	GE 1.5T	Three-dimensional T1-weighted images, using a spoiled grass (SPGR) sequence acquired in the coronal plane with: echo time (TE)=5 ms, repetition time (TR)=24 ms,	Coronal	v5.0.0	Ubuntu 11,04 (x86_64)	1 subject was excluded because motion artifacts resulted in very poor segmentation

			numbers of excitations (NEX)=2, rotation angle=45°, field of view (FOV)=26×19.5 cm, slice thickness=1.5mm and a matrix of 256×192.				
**PAFIP3T**	1						
**RSCZ**	1	3T Philips Achieva	A turbo field echo sequence covering the whole brain. TR = 8,200 ms, TE = 3.7 ms, TI = 1,020 ms, flip angle = 8, SENSE factor = 1.5, FOV = 240 mm, voxel size of 0.83 × 0.83 mm with a slice thickness of 1 mm, no gap.	Sagittal	v5.3.0	Centos 6.6	
**RomeSL**	1	3T Siemens Allegra	T1-weighted, 3D MDEFT, 1x1x1xmm, TE/TR =2.4/7.92 ms, flip angle=15	Sagittal	6.0dev	linux	
**SCORE**	1		MPRAGE: aquicition matrix: 256×256×176, isotropic spatial resolution: 1x1x1mm3, TI=1000ms, TR=2s, TE=3.4 ms, flip angle: 8° and bandwidth of 200 Hz/pixel	Sagittal	6.0dev		

**SNUH**	1	3T Siemens Trio	high-resolution T1-weighted, three-dimensional Magnetization Prepared Rapid Gradient Echo (TR = 670ms; TE=1.89ms; FOV=250mm; FA=9°; voxel size=1x1x1mm3)	Sagittal	v5.3.0	OSX 10.9	
**SaoPaolo**	1	1.5T GE Signa	T1-SPGR (fast spoiled gradient recall sequence) with 124 contiguous slices, voxel size 0.8660.8661.5 mm, echo time 5.2ms, repitition time 21.7ms, flip angle=20, field of view=22, matrix 256x256x192	Axial	v5.3.0	OSX	The skull-stripping was performed using a automated method known as Multi-Atlas Skull-Stripping (MASS version 1.0), followed by manual corrections. This step was done separately from the FreeSurfer pipeline (automatic reconstruction). No subject was removed due to Q&A.
**TOP**	1	1.5T Siemens Magnetom Sonata	Two sagittal T1-weighted magnetization prepared rapid gradient echo (MPRAGE) volumes were acquired with the Siemens tfl3d1_ns pulse sequence (TE = 3.93 ms, TR = 2730 ms, TI = 1000 ms, flip angle = 7°; FOV = 24 cm, voxel size= 1.33 x 0.94 x 1 mm3, number of partitions = 160)	Sagittal	v4.5.0	Linux Centos or Ubuntu	0

**UMCU**	1	Philips 1.5T Intera and Achieva	T1-weighted three-dimensional fast-field echo (3D-FFE) scans with 160–180 contiguous coronal slices [256 3 256 matrix, echo time (TE)=4.6 ms, repetition time (TR)=30 ms, flip angle=30 degrees, 1x1x1.2 mm3 voxels, field of view [FOV] = 256 mm/ 70%]	Coronal	v5.1.0	Linux-centos4_x86_64-stable-pub	0
**UMCUS**	1	3T Philips Achieva	T1-weighted 3D FFE, TR/TE 9.86/4.6ms, 0.875x0.875x1 voxels, flip angle 8, FOV 224x160x168, 160 slices	Axial	v5.3.0	Ubuntu 10.04, Kernel Linux 2.6.32-25-generic, GNOME 2.30.2	
**UNIBA**	1	3T GE	T1-weighted 3D FFE, TR/TE 9.86/4.6ms, 0.875x0.875x1 voxels, flip angle 8, FOV 224x160x168, 160 slices	Axial	v5.3.0	Ubuntu 10.04, Kernel Linux 2.6.32-25-generic, GNOME 2.30.2	

**UPENN**	2	Siemens 3T	MPRAGE, TR=1810 ms, TE= 3.51 ms, TI=1100 ms, flip angle 9, FOV= 240 x 180 mm, matrix= 256 × 192, resolution = 0.9 x 0.9 mm, slices = 160, slice/skip thickness = 1 mm/0 mm	Axial	v5.3.0	Linux Red Hat Enterprise 5	0

Table S6. Absolute Means (SD) of Cortical Thickness in ENIGMA SCZ sample adapted from van Erp et al.[1]

	Cortical Thickness of entire ENIGMA SCZ sample		
	Weighted Mean	Pooled SD	N
Left banks of superior temporal sulcus	2.408	0.003	4350
Left caudal anterior cingulate cortex	2.626	0.004	4430
Left caudal middle frontal gyrus	2.485	0.003	4417
Left cuneus	1.808	0.002	4361
Left entorhinal cortex	3.234	0.005	4311
Left fusiform gyrus	2.591	0.003	4375
Left inferior parietal cortex	2.39	0.002	4315
Left inferior temporal gyrus	2.677	0.003	4351
Left isthmus cingulate cortex	2.449	0.003	4428
Left lateral occipital cortex	2.124	0.002	4376
Left lateral orbitofrontal cortex	2.621	0.003	4431
Left lingual gyrus	1.954	0.002	4427
Left medial orbitofrontal cortex	2.457	0.003	4425
Left middle temporal gyrus	2.788	0.003	4312
Left parahippocampal gyrus	2.604	0.005	4410
Left paracentral lobule	2.302	0.003	4428
Left pars opercularis of inferior frontal gyrus	2.529	0.003	4395
Left pars orbitalis of inferior frontal gyrus	2.693	0.004	4399
Left pars triangularis of inferior frontal gyrus	2.44	0.003	4379
Left pericalcarine cortex	1.595	0.002	4435
Left postcentral gyrus	2.019	0.002	4419
Left posterior cingulate cortex	2.506	0.003	4431
Left precentral gyrus	2.446	0.002	4415
Left precuneus	2.301	0.002	4408
Left rostral anterior cingulate cortex	2.835	0.004	4423
Left rostral middle frontal gyrus	2.367	0.002	4383
Left superior frontal gyrus	2.703	0.003	4423
Left superior parietal cortex	2.132	0.002	4361
Left superior temporal gyrus	2.708	0.003	4295
Left supramarginal gyrus	2.472	0.002	4292
Left frontal pole	2.801	0.005	4437

Left temporal pole	3.576	0.005	4394
Left transverse temporal gyrus	2.29	0.004	4435
Left insula	3.002	0.003	4431
Right banks of superior temporal sulcus	2.496	0.003	4382
Right caudal anterior cingulate cortex	2.547	0.004	4427
Right caudal middle frontal gyrus	2.474	0.003	4405
Right cuneus	1.827	0.002	4386
Right entorhinal cortex	3.382	0.006	4217
Right fusiform gyrus	2.596	0.003	4347
Right inferior parietal cortex	2.411	0.002	4306
Right inferior temporal gyrus	2.701	0.003	4345
Right isthmus cingulate cortex	2.403	0.003	4414
Right lateral occipital cortex	2.169	0.002	4361
Right lateral orbitofrontal cortex	2.594	0.003	4401
Right lingual gyrus	1.996	0.002	4426
Right medial orbitofrontal cortex	2.431	0.003	4388
Right middle temporal gyrus	2.825	0.003	4327
Right parahippocampal gyrus	2.583	0.004	4385
Right paracentral lobule	2.318	0.002	4425
Right pars opercularis of inferior frontal gyrus	2.531	0.003	4372
Right pars orbitalis of inferior frontal gyrus	2.675	0.003	4401
Right pars triangularis of inferior frontal gyrus	2.449	0.003	4373
Right pericalcarine cortex	1.602	0.002	4427
Right postcentral gyrus	1.993	0.002	4408
Right posterior cingulate cortex	2.481	0.003	4428
Right precentral gyrus	2.419	0.002	4404
Right precuneus	2.313	0.002	4398
Right rostral anterior cingulate cortex	2.833	0.004	4397
Right rostral middle frontal gyrus	2.341	0.002	4372
Right superior frontal gyrus	2.683	0.003	4408
Right superior parietal cortex	2.123	0.002	4382
Right superior temporal gyrus	2.738	0.003	4327
Right supramarginal gyrus	2.478	0.002	4297
Right frontal pole	2.763	0.005	4431
Right temporal pole	3.683	0.006	4294

Right transverse temporal gyrus	2.328	0.004	4430
Right insula	2.977	0.003	4427

Fig. S1 OFC covariance networks derived from mean cortical thickness data of 4474 schizophrenia individuals from the ENIGMA consortium[1]

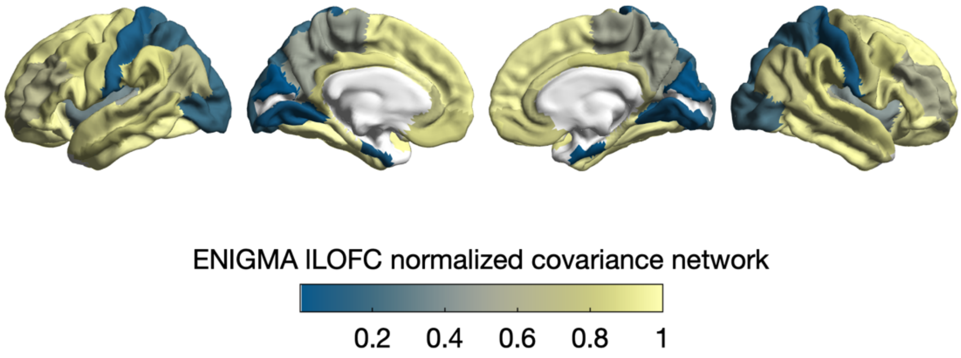

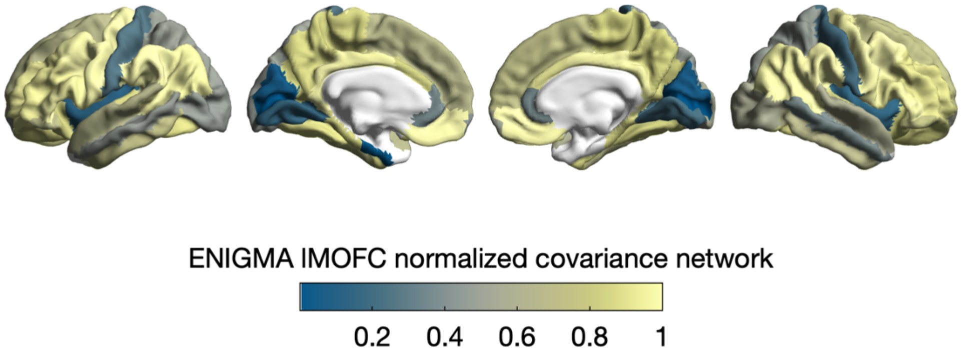

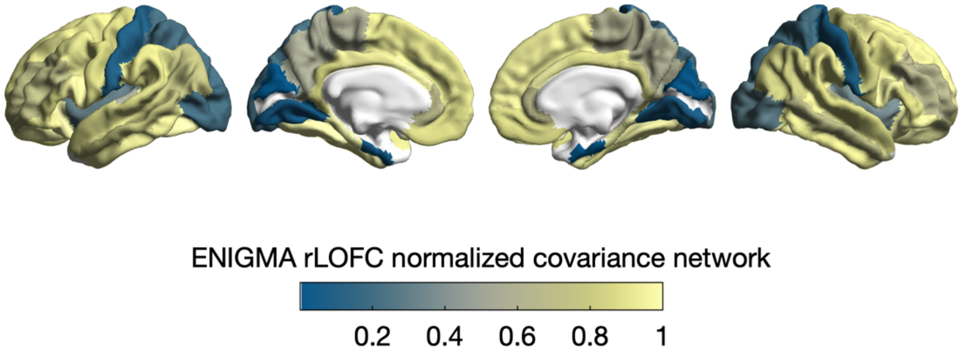
ENIGMA rLOFC normalized covariance network
0.2
0.4
0.6
0.8
1

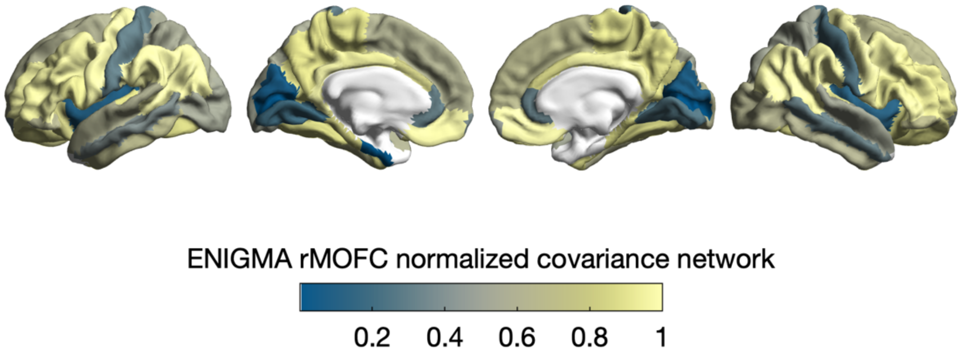
ENIGMA rMOFC normalized covariance network
0.2
0.4
0.6
0.8
1

Fig. S2. Association between cortical thickness and negative symptom severity in schizophrenia individuals from the ENIGMA consortium[1]

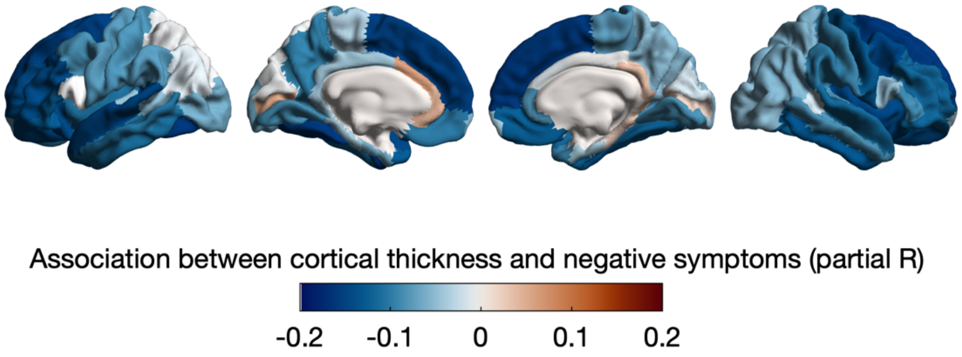

Fig. S2. For each DK atlas region (n=68), partial correlation between negative symptom severity and cortical thickness correcting for age, and sex were performed using R (version 3.2.2) metafor package (version 1.9-7). Analyses were performed for subsamples with PANSS negative scores (n=1926), and subsamples with SANS total scores (n=1588) separately. Effect sizes from both analyses were combined in one average effect size map displaying the partial R for each cortical region.

Table S7a. Correlations of negative symptoms and OFC thickness across the BD-SCZ spectrum

Region	Amotivation	FDR adjusted p-value	DimEx	FDR adjusted p-value
L LOFC	-0.121	0.240	-0.194	0.223
R LOFC	-0.346	0.002	-0.135	0.264
L MOFC	-0.289	0.008	-0.131	0.264
R MOFC	-0.221	0.039	-0.090	0.380

Table S7a. FDR adjusted p value, false discovery rate adjusted p value; DimEx, diminished expression; LOFC, lateral orbitofrontal cortex; MOFC, medial orbitofrontal cortex; L, left; R, right

Table S7b. Correlation amotivation and OFC in BD and SCZ separately

Region	BD Amotivation	p-value	SCZ Amotivation	p-value
L LOFC	-0.118	0.42	-0.107	0.464
R LOFC	**-0.371**	0.009	**-0.323**	0.024
L MOFC	**-0.322**	0.026	**-0.342**	0.016
R MOFC	**-0.298**	0.040	**-0.239**	0.099

Table S7b. BD, bipolar disorder; SCZ, schizophrenia; LOFC, lateral orbitofrontal cortex; MOFC, medial orbitofrontal cortex; L, left; R, right.

Table S8. Correlations of amotivation and diminished expression with striatal subregions across BD-SCZ

Structures	Amotivation	p value	Diminished expression	p value
L accumbens	-0.055	0.589	-0.010	0.921
R accumbens	-0.002	0.987	0.007	0.943
L caudate	0.113	0.266	0.102	0.320
R caudate	0.054	0.596	0.073	0.474
L putamen	0.009	0.932	0.179	0.078
R putamen	0.034	0.739	0.112	0.274

Table S9. Correlations between other clinical factors and OFC across the BD-SCZ spectrum

Region	Risp eq dose	p-value	Positive symptoms	p-value	Disorganization	p-value	HAMD-21	p-value
L LOFC	**-0.280**	0.005	0.016	0.687	-0.022	0.755	0.053	0.365
R LOFC	**-0.201**	0.046	0.034	0.560	0.022	0.814	-0.069	0.610
L MOFC	-0.062	0.476	0.017	0.747	-.132	0.242	-0.021	0.799
R MOFC	0.010	0.959	-0.016	0.820	-0.161	0.144	-0.154	0.223

Table S9. risp eq dose, risperidone equivalent dose, LOFC, lateral orbitofrontal cortex; MOFC, medial orbitofrontal cortex; L, left; R, right

Fig S3. Normative functional degree centrality derived from the HCP sample (n = 207)[2–4]

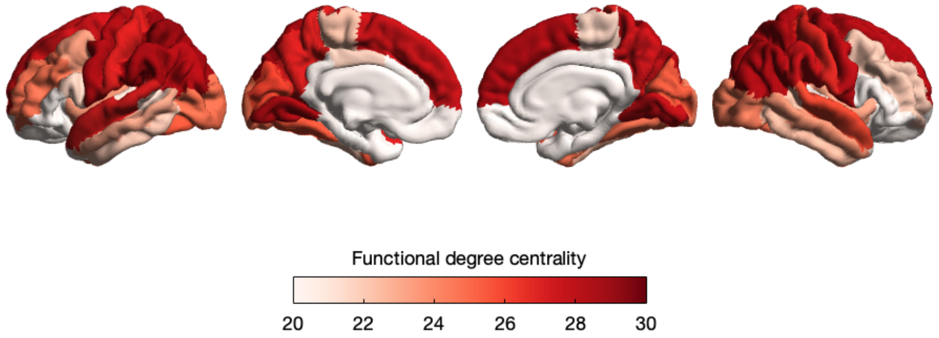

Fig S4. Normative structural degree centrality derived from the HCP sample (n = 207)[2–4]

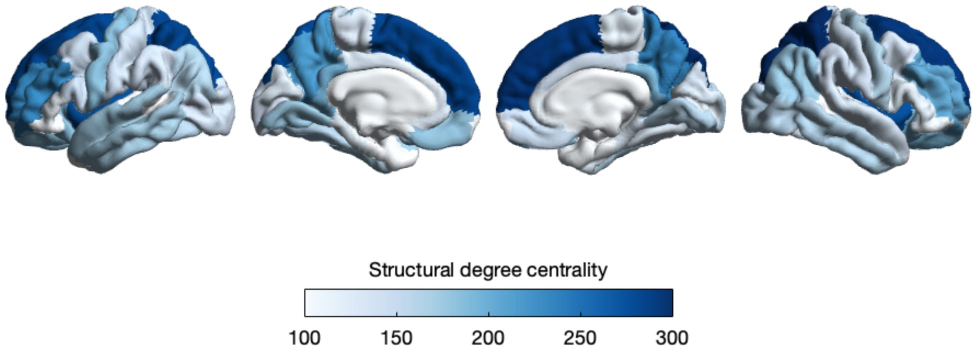

Fig. S5. Association between covariance networks and cortical effect sizes (correlation r) of amotivation and diminished expression across the BD-SCZ spectrum

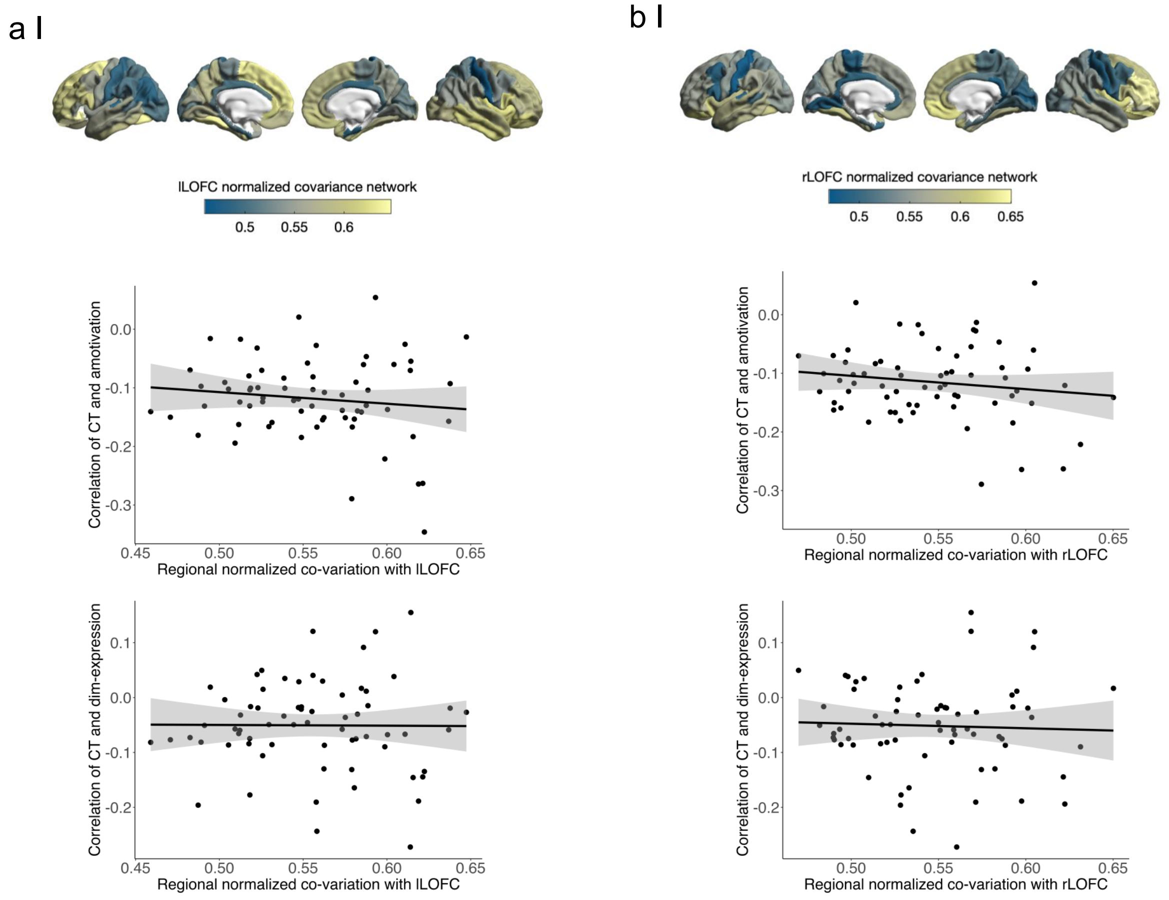

Figure S5. Association between covariance networks and cortical effect sizes (correlation r) of amotivation and diminished expression across the BD-SCZ spectrum. **(a)** Correlations between the left lOFC co-variations network and the cortical effect size maps of amotivation and diminished expression respectively. The left lOFC covariance network did not show significant spatially correlation with the cortical alteration pattern of amotivation or diminished expression (amotivation, rs = -0.080, p_{spin} = 0.29; diminished expression, rs = -0.003, p_{spin} = 0.47). **(b)** Correlations between the right lOFC covariance network and the cortical effect size maps of amotivation and diminished expression respectively. Similar to the results with the left lOFC covariance network, the right lOFC covariance network did not show significant correlations with the cortical alteration pattern of amotivation or diminished expression (amotivation, rs = -0.090, p_{spin} = 0.27; diminished expression, rs = -0.06, p_{spin} = 0.31).

Table S10. Spatial correlation of covariance networks with amotivation-related cortical alterations

DKT_Region	r_value	pspin	Rank
L_medialorbitofrontal_thickavg	-0.425	0.001	1
R_frontalpole_thickavg	-0.285	0.010	2
R_caudalanteriorcingulate_thickavg	-0.202	0.053	3
R_medialorbitofrontal_thickavg	-0.163	0.089	4
R_insula_thickavg	-0.142	0.113	5
L_inferiorparietal_thickavg	-0.127	0.159	6
R_entorhinal_thickavg	-0.124	0.160	7
R_parsopercularis_thickavg	-0.122	0.197	8
R_cuneus_thickavg	-0.111	0.195	9
L_frontalpole_thickavg	-0.100	0.201	10
R_pericalcarine_thickavg	-0.086	0.195	11
R_lateralorbitofrontal_thickavg	-0.085	0.272	12
L_lateralorbitofrontal_thickavg	-0.078	0.290	13
R_postcentral_thickavg	-0.076	0.257	14
L_rostralanteriorcingulate_thickavg	-0.074	0.273	15
L_cuneus_thickavg	-0.061	0.327	16
R_lingual_thickavg	-0.053	0.279	17
L_lateraloccipital_thickavg	-0.045	0.370	18
L_superiorparietal_thickavg	-0.027	0.395	19
R_rostralmiddlefrontal_thickavg	-0.024	0.463	20
R_transversetemporal_thickavg	-0.022	0.423	21
L_transversetemporal_thickavg	0.001	0.567	22
L_caudalanteriorcingulate_thickavg	0.004	0.442	23
L_insula_thickavg	0.004	0.459	24
R_precuneus_thickavg	0.005	0.464	25
L_lingual_thickavg	0.005	0.487	26
R_parstriangularis_thickavg	0.010	0.437	27
R_supramarginal_thickavg	0.011	0.474	28
L_postcentral_thickavg	0.017	0.459	29
L_paracentral_thickavg	0.019	0.450	30
R_rostralanteriorcingulate_thickavg	0.020	0.396	31
L_parsorbitalis_thickavg	0.021	0.411	32
L_parsopercularis_thickavg	0.027	0.400	33
L_superiortemporal_thickavg	0.029	0.418	34
L_precuneus_thickavg	0.034	0.381	35
R_superiorfrontal_thickavg	0.036	0.340	36
R_paracentral_thickavg	0.038	0.343	37
R_superiorparietal_thickavg	0.042	0.405	38
R_inferiortemporal_thickavg	0.045	0.336	39
L_pericalcarine_thickavg	0.047	0.348	40
L_isthmuscingulate_thickavg	0.049	0.326	41
R_parahippocampal_thickavg	0.052	0.381	42
R_inferiorparietal_thickavg	0.052	0.339	43
L_entorhinal_thickavg	0.052	0.328	44
L_rostralmiddlefrontal_thickavg	0.052	0.352	45
R_superiortemporal_thickavg	0.053	0.350	46
L_superiorfrontal_thickavg	0.057	0.346	47
R_parsorbitalis_thickavg	0.057	0.264	48
L_supramarginal_thickavg	0.059	0.277	49

R_lateraloccipital_thickavg	0.068	0.322	50
L_posteriorcingulate_thickavg	0.070	0.243	51
L_caudalmiddlefrontal_thickavg	0.072	0.271	52
L_parahippocampal_thickavg	0.078	0.237	53
R_precentral_thickavg	0.088	0.169	54
L_inferiortemporal_thickavg	0.094	0.207	55
L_parstriangularis_thickavg	0.100	0.215	56
R_caudalmiddlefrontal_thickavg	0.110	0.168	57
R_posteriorcingulate_thickavg	0.121	0.128	58
R_bankssts_thickavg	0.128	0.128	59
R_fusiform_thickavg	0.128	0.135	60
R_temporalpole_thickavg	0.147	0.108	61
L_precentral_thickavg	0.151	0.080	62
R_middletemporal_thickavg	0.163	0.090	63
L_bankssts_thickavg	0.163	0.101	64
R_isthmuscingulate_thickavg	0.194	0.054	65
L_fusiform_thickavg	0.198	0.045	66
L_middletemporal_thickavg	0.216	0.031	67
L_temporalpole_thickavg	0.272	0.016	68

Table S11. Spatial correlation of covariance networks with diminished expression-related cortical alterations

DKT_Region	r_value	pspin	Rank
L_medialorbitofrontal_thickavg	-0.385	0.004	1
R_entorhinal_thickavg	-0.301	0.017	2
R_insula_thickavg	-0.223	0.042	3
L_frontalpole_thickavg	-0.209	0.056	4
L_transversetemporal_thickavg	-0.145	0.142	5
R_medialorbitofrontal_thickavg	-0.131	0.195	6
L_rostralanteriorcingulate_thickavg	-0.120	0.158	7
R_frontalpole_thickavg	-0.108	0.212	8
L_isthmuscingulate_thickavg	-0.097	0.195	9
R_rostralanteriorcingulate_thickavg	-0.097	0.240	10
R_lateralorbitofrontal_thickavg	-0.059	0.311	11
L_posteriorcingulate_thickavg	-0.058	0.299	12
L_parahippocampal_thickavg	-0.049	0.330	13
R_transversetemporal_thickavg	-0.041	0.370	14
R_inferiortemporal_thickavg	-0.021	0.443	15
L_lateralorbitofrontal_thickavg	-0.003	0.469	16
L_insula_thickavg	0.001	0.462	17
L_caudalanteriorcingulate_thickavg	0.010	0.469	18
L_inferiorparietal_thickavg	0.018	0.441	19
L_entorhinal_thickavg	0.022	0.426	20
R_fusiform_thickavg	0.024	0.420	21
R_isthmuscingulate_thickavg	0.029	0.419	22
L_parsorbitalis_thickavg	0.049	0.363	23
L_cuneus_thickavg	0.053	0.384	24
L_inferiortemporal_thickavg	0.054	0.358	25
R_precuneus_thickavg	0.071	0.314	26
R_superiortemporal_thickavg	0.076	0.289	27
R_middletemporal_thickavg	0.078	0.248	28
R_caudalanteriorcingulate_thickavg	0.081	0.260	29
L_precuneus_thickavg	0.088	0.255	30
R_parahippocampal_thickavg	0.088	0.237	31
L_fusiform_thickavg	0.106	0.195	32
L_superiortemporal_thickavg	0.110	0.194	33
R_rostralmiddlefrontal_thickavg	0.118	0.196	34
R_pericalcarine_thickavg	0.118	0.178	35
L_postcentral_thickavg	0.119	0.187	36
L_superiorfrontal_thickavg	0.121	0.164	37
R_lateraloccipital_thickavg	0.122	0.157	38
L_supramarginal_thickavg	0.129	0.142	39
R_inferiorparietal_thickavg	0.135	0.125	40
L_parsopercularis_thickavg	0.137	0.137	41
R_supramarginal_thickavg	0.150	0.103	42
L_lateraloccipital_thickavg	0.151	0.120	43
R_bankssts_thickavg	0.152	0.114	44
R_lingual_thickavg	0.155	0.135	45

R_parsopercularis_thickavg	0.156	0.135	46
L_pericalcarine_thickavg	0.158	0.102	47
R_postcentral_thickavg	0.166	0.111	48
R_temporalpole_thickavg	0.166	0.115	49
L_superiorparietal_thickavg	0.166	0.096	50
R_superiorparietal_thickavg	0.173	0.074	51
R_posteriorcingulate_thickavg	0.183	0.080	52
L_temporalpole_thickavg	0.183	0.079	53
L_caudalmiddlefrontal_thickavg	0.186	0.076	54
L_lingual_thickavg	0.188	0.098	55
L_parstriangularis_thickavg	0.191	0.069	56
L_middletemporal_thickavg	0.194	0.062	57
L_bankssts_thickavg	0.194	0.049	58
R_cuneus_thickavg	0.207	0.064	59
L_rostralmiddlefrontal_thickavg	0.217	0.038	60
R_parstriangularis_thickavg	0.236	0.037	61
R_parsorbitalis_thickavg	0.248	0.024	62
R_precentral_thickavg	0.257	0.037	63
R_superiorfrontal_thickavg	0.263	0.014	64
L_paracentral_thickavg	0.266	0.024	65
L_precentral_thickavg	0.278	0.017	66
R_paracentral_thickavg	0.286	0.018	67
R_caudalmiddlefrontal_thickavg	0.341	0.004	68

Table S12. Functional epicenters of amotivation-related cortical alteration pattern

DKT_Region	r_value	pspin	Rank
R_rostralanteriorcingulate_thickavg	-0.295	0.009	1
R_posteriorcingulate_thickavg	-0.243	0.025	2
L_rostralanteriorcingulate_thickavg	-0.232	0.024	3
L_transversetemporal_thickavg	-0.193	0.046	4
L_caudalanteriorcingulate_thickavg	-0.185	0.057	5
R_caudalanteriorcingulate_thickavg	-0.180	0.065	6
L_posteriorcingulate_thickavg	-0.151	0.082	7
R_supramarginal_thickavg	-0.124	0.154	8
L_lateralorbitofrontal_thickavg	-0.117	0.089	9
R_transversetemporal_thickavg	-0.113	0.165	10
R_medialorbitofrontal_thickavg	-0.107	0.173	11
R_superiortemporal_thickavg	-0.104	0.171	12
R_precentral_thickavg	-0.103	0.170	13
R_superiorfrontal_thickavg	-0.100	0.212	14
L_insula_thickavg	-0.095	0.216	15
R_parstriangularis_thickavg	-0.090	0.256	16
L_postcentral_thickavg	-0.080	0.234	17
L_isthmuscingulate_thickavg	-0.079	0.232	18
L_parsopercularis_thickavg	-0.075	0.184	19
L_paracentral_thickavg	-0.070	0.279	20
L_superiortemporal_thickavg	-0.068	0.259	21
R_temporalpole_thickavg	-0.067	0.297	22
L_parahippocampal_thickavg	-0.066	0.266	23
R_insula_thickavg	-0.065	0.302	24
R_paracentral_thickavg	-0.063	0.262	25
L_pericalcarine_thickavg	-0.062	0.293	26
R_isthmuscingulate_thickavg	-0.055	0.301	27
R_postcentral_thickavg	-0.053	0.302	28
R_precuneus_thickavg	-0.051	0.329	29
L_precentral_thickavg	-0.051	0.321	30
L_precuneus_thickavg	-0.042	0.336	31
R_rostralmiddlefrontal_thickavg	-0.040	0.433	32
R_parahippocampal_thickavg	-0.035	0.357	33
L_frontalpole_thickavg	-0.032	0.308	34
R_pericalcarine_thickavg	-0.025	0.360	35
R_middletemporal_thickavg	-0.024	0.421	36
L_superiorparietal_thickavg	-0.023	0.448	37
R_cuneus_thickavg	-0.012	0.442	38
L_superiorfrontal_thickavg	-0.005	0.384	39

L_rostralmiddlefrontal_thickavg	-0.003	0.443	40
R_superiorparietal_thickavg	-0.001	0.482	41
R_caudalmiddlefrontal_thickavg	0.000	0.467	42
L_lingual_thickavg	0.001	0.503	43
L_lateraloccipital_thickavg	0.008	0.460	44
R_lingual_thickavg	0.009	0.485	45
R_lateralorbitofrontal_thickavg	0.011	0.378	46
L_bankssts_thickavg	0.014	0.545	47
R_frontalpole_thickavg	0.018	0.445	48
L_medialorbitofrontal_thickavg	0.018	0.519	49
R_parsopercularis_thickavg	0.020	0.397	50
L_cuneus_thickavg	0.029	0.382	51
R_bankssts_thickavg	0.030	0.411	52
L_parstriangularis_thickavg	0.032	0.494	53
R_fusiform_thickavg	0.038	0.381	54
R_entorhinal_thickavg	0.039	0.335	55
R_parsorbitalis_thickavg	0.043	0.350	56
R_lateraloccipital_thickavg	0.051	0.378	57
L_caudalmiddlefrontal_thickavg	0.057	0.422	58
L_fusiform_thickavg	0.060	0.304	59
L_parsorbitalis_thickavg	0.062	0.392	60
L_temporalpole_thickavg	0.069	0.380	61
L_entorhinal_thickavg	0.070	0.375	62
R_inferiorparietal_thickavg	0.073	0.246	63
L_inferiorparietal_thickavg	0.075	0.319	64
R_inferiortemporal_thickavg	0.080	0.229	65
L_inferiortemporal_thickavg	0.083	0.293	66
L_supramarginal_thickavg	0.086	0.242	67
L_middletemporal_thickavg	0.189	0.078	68

Table S13. Structural epicenters of amotivation-related cortical alteration pattern

DKT_Region	r_value	pspin	Rank
R_rostralanteriorcingulate_thickavg	-0.410	0.001	1
R_frontalpole_thickavg	-0.358	0.002	2
R_caudalanteriorcingulate_thickavg	-0.348	0.006	3
L_frontalpole_thickavg	-0.347	0.011	4
R_superiorfrontal_thickavg	-0.313	0.005	5
R_medialorbitofrontal_thickavg	-0.275	0.018	6
L_rostralanteriorcingulate_thickavg	-0.274	0.002	7
R_parsorbitalis_thickavg	-0.265	0.043	8
L_lateralorbitofrontal_thickavg	-0.251	0.003	9
L_caudalanteriorcingulate_thickavg	-0.219	0.014	10
R_temporalpole_thickavg	-0.211	0.101	11
R_posteriorcingulate_thickavg	-0.200	0.057	12
R_parstriangularis_thickavg	-0.199	0.112	13
R_lateralorbitofrontal_thickavg	-0.188	0.109	14
R_rostralmiddlefrontal_thickavg	-0.171	0.121	15
L_superiorfrontal_thickavg	-0.167	0.051	16
L_posteriorcingulate_thickavg	-0.158	0.068	17
R_precentral_thickavg	-0.123	0.224	18
L_parsorbitalis_thickavg	-0.122	0.040	19
R_insula_thickavg	-0.121	0.372	20
R_caudalmiddlefrontal_thickavg	-0.116	0.219	21
L_medialorbitofrontal_thickavg	-0.111	0.117	22
R_parsopercularis_thickavg	-0.106	0.287	23
R_superiorparietal_thickavg	-0.073	0.379	24
R_postcentral_thickavg	-0.061	0.351	25
R_isthmuscingulate_thickavg	-0.058	0.435	26
L_precuneus_thickavg	-0.057	0.200	27
R_pericalcarine_thickavg	-0.054	0.378	28
R_middletemporal_thickavg	-0.054	0.555	29
R_precuneus_thickavg	-0.052	0.460	30
L_bankssts_thickavg	-0.035	0.237	31
R_supramarginal_thickavg	-0.033	0.524	32
L_transversetemporal_thickavg	-0.033	0.241	33
L_temporalpole_thickavg	-0.011	0.259	34
R_paracentral_thickavg	-0.010	0.430	35
R_parahippocampal_thickavg	-0.009	0.676	36
L_caudalmiddlefrontal_thickavg	-0.005	0.373	37
R_transversetemporal_thickavg	-0.003	0.561	38
L_rostralmiddlefrontal_thickavg	0.000	0.630	39
R_superiortemporal_thickavg	0.010	0.287	40
L_postcentral_thickavg	0.011	0.538	41
L_parsopercularis_thickavg	0.014	0.650	42
L_lingual_thickavg	0.019	0.616	43
L_parstriangularis_thickavg	0.022	0.595	44
R_lingual_thickavg	0.023	0.290	45
L_paracentral_thickavg	0.030	0.417	46
L_insula_thickavg	0.030	0.771	47
L_superiorparietal_thickavg	0.033	0.547	48
L_isthmuscingulate_thickavg	0.039	0.468	49

R_fusiform_thickavg	0.046	0.183	50
R_inferiorparietal_thickavg	0.047	0.166	51
L_precentral_thickavg	0.052	0.438	52
R_cuneus_thickavg	0.058	0.266	53
L_parahippocampal_thickavg	0.060	0.526	54
L_inferiorparietal_thickavg	0.075	0.421	55
R_lateraloccipital_thickavg	0.075	0.150	56
L_pericalcarine_thickavg	0.084	0.310	57
L_cuneus_thickavg	0.087	0.271	58
R_entorhinal_thickavg	0.088	0.131	59
L_entorhinal_thickavg	0.104	0.275	60
L_superiortemporal_thickavg	0.113	0.320	61
L_inferiortemporal_thickavg	0.136	0.208	62
R_inferiortemporal_thickavg	0.140	0.048	63
R_bankssts_thickavg	0.160	0.047	64
L_supramarginal_thickavg	0.183	0.091	65
L_lateraloccipital_thickavg	0.218	0.048	66
L_fusiform_thickavg	0.221	0.040	67
L_middletemporal_thickavg	0.222	0.045	68

Table S14. Functional epicenters of diminished expression-related cortical alteration pattern

DKT_Region	r_value	pspin	Rank
R_rostralanteriorcingulate_thickavg	-0.172	0.096	1
R_temporalpole_thickavg	-0.118	0.184	2
L_rostralanteriorcingulate_thickavg	-0.103	0.211	3
L_medialorbitofrontal_thickavg	-0.093	0.225	4
R_superiortemporal_thickavg	-0.082	0.252	5
R_medialorbitofrontal_thickavg	-0.072	0.258	6
R_posteriorcingulate_thickavg	-0.070	0.285	7
L_insula_thickavg	-0.042	0.342	8
L_parstriangularis_thickavg	-0.034	0.385	9
R_precentral_thickavg	-0.033	0.377	10
L_transversetemporal_thickavg	-0.027	0.414	11
L_parahippocampal_thickavg	-0.025	0.408	12
R_frontalpole_thickavg	-0.024	0.433	13
L_entorhinal_thickavg	-0.022	0.413	14
L_temporalpole_thickavg	-0.016	0.474	15
L_lateraloccipital_thickavg	-0.016	0.425	16
L_isthmuscingulate_thickavg	-0.014	0.420	17
R_caudalanteriorcingulate_thickavg	-0.008	0.468	18
R_superiorparietal_thickavg	-0.007	0.483	19
L_precuneus_thickavg	-0.005	0.465	20
R_parsorbitalis_thickavg	-0.003	0.465	21
L_posteriorcingulate_thickavg	-0.001	0.480	22
L_fusiform_thickavg	0.001	0.520	23
R_isthmuscingulate_thickavg	0.003	0.501	24
R_superiorfrontal_thickavg	0.015	0.473	25
R_parahippocampal_thickavg	0.017	0.458	26
R_supramarginal_thickavg	0.018	0.459	27
R_paracentral_thickavg	0.018	0.455	28
L_caudalmiddlefrontal_thickavg	0.023	0.420	29
L_superiortemporal_thickavg	0.024	0.449	30
R_fusiform_thickavg	0.025	0.440	31
R_rostralmiddlefrontal_thickavg	0.026	0.424	32
L_postcentral_thickavg	0.027	0.468	33
R_parstriangularis_thickavg	0.027	0.414	34
R_precuneus_thickavg	0.028	0.411	35
L_precentral_thickavg	0.029	0.432	36
R_insula_thickavg	0.031	0.425	37
L_caudalanteriorcingulate_thickavg	0.033	0.415	38
L_superiorfrontal_thickavg	0.038	0.349	39
L_parsopercularis_thickavg	0.040	0.348	40

L_paracentral_thickavg	0.042	0.401	41
L_inferiortemporal_thickavg	0.045	0.314	42
R_bankssts_thickavg	0.049	0.356	43
L_pericalcarine_thickavg	0.049	0.369	44
L_frontalpole_thickavg	0.051	0.310	45
R_postcentral_thickavg	0.056	0.322	46
R_entorhinal_thickavg	0.057	0.345	47
L_supramarginal_thickavg	0.063	0.342	48
R_inferiorparietal_thickavg	0.065	0.301	49
L_cuneus_thickavg	0.066	0.326	50
R_cuneus_thickavg	0.077	0.287	51
L_superiorparietal_thickavg	0.078	0.302	52
R_middletemporal_thickavg	0.083	0.259	53
L_bankssts_thickavg	0.087	0.252	54
L_rostralmiddlefrontal_thickavg	0.088	0.223	55
R_transversetemporal_thickavg	0.092	0.246	56
L_parsorbitalis_thickavg	0.099	0.188	57
R_lateraloccipital_thickavg	0.100	0.205	58
L_inferiorparietal_thickavg	0.102	0.209	59
R_pericalcarine_thickavg	0.108	0.200	60
R_lateralorbitofrontal_thickavg	0.112	0.206	61
L_lateralorbitofrontal_thickavg	0.116	0.172	62
R_lingual_thickavg	0.121	0.177	63
L_middletemporal_thickavg	0.126	0.129	64
R_caudalmiddlefrontal_thickavg	0.133	0.158	65
L_lingual_thickavg	0.135	0.162	66
R_parsopercularis_thickavg	0.161	0.102	67
R_inferiortemporal_thickavg	0.205	0.054	68

Table S15. Structural epicenters of diminished expression-related cortical alteration pattern

DKT_Region	r_value	pspin
R_temporalpole_thickavg	-0.348	0.005
L_medialorbitofrontal_thickavg	-0.217	0.045
R_frontalpole_thickavg	-0.178	0.080
L_rostralanteriorcingulate_thickavg	-0.177	0.067
R_rostralanteriorcingulate_thickavg	-0.151	0.131
R_caudalanteriorcingulate_thickavg	-0.133	0.157
L_bankssts_thickavg	-0.122	0.124
R_parsorbitalis_thickavg	-0.117	0.167
R_fusiform_thickavg	-0.103	0.229
L_caudalanteriorcingulate_thickavg	-0.094	0.188
R_superiortemporal_thickavg	-0.090	0.240
R_parahippocampal_thickavg	-0.075	0.265
R_medialorbitofrontal_thickavg	-0.065	0.278
L_frontalpole_thickavg	-0.060	0.306
L_posteriorcingulate_thickavg	-0.057	0.346
R_superiorparietal_thickavg	-0.051	0.302
R_bankssts_thickavg	-0.041	0.324
R_superiorfrontal_thickavg	-0.038	0.374
R_supramarginal_thickavg	-0.038	0.361
R_inferiorparietal_thickavg	-0.026	0.412
L_lateralorbitofrontal_thickavg	-0.024	0.456
R_lingual_thickavg	-0.023	0.414
R_insula_thickavg	-0.022	0.356
R_parstriangularis_thickavg	-0.019	0.429
R_posteriorcingulate_thickavg	-0.010	0.427
L_parstriangularis_thickavg	-0.003	0.511
R_entorhinal_thickavg	-0.002	0.480
L_temporalpole_thickavg	0.000	0.474
R_cuneus_thickavg	0.009	0.494
L_superiorfrontal_thickavg	0.014	0.472
R_lateralorbitofrontal_thickavg	0.022	0.437
L_transversetemporal_thickavg	0.022	0.412
R_middletemporal_thickavg	0.024	0.448
R_transversetemporal_thickavg	0.027	0.458
L_precuneus_thickavg	0.031	0.393
R_isthmuscingulate_thickavg	0.036	0.389
R_pericalcarine_thickavg	0.040	0.390
R_inferiortemporal_thickavg	0.041	0.396
L_parsorbitalis_thickavg	0.042	0.314
L_lateraloccipital_thickavg	0.043	0.313
L_caudalmiddlefrontal_thickavg	0.044	0.350
L_entorhinal_thickavg	0.047	0.300
R_rostralmiddlefrontal_thickavg	0.047	0.364
L_parahippocampal_thickavg	0.057	0.234
R_precuneus_thickavg	0.062	0.327
R_caudalmiddlefrontal_thickavg	0.063	0.346
L_insula_thickavg	0.063	0.185
L_supramarginal_thickavg	0.065	0.219

L_parsopercularis_thickavg	0.070	0.209
R_precentral_thickavg	0.070	0.326
L_inferiorparietal_thickavg	0.071	0.232
L_isthmuscingulate_thickavg	0.075	0.288
L_middletemporal_thickavg	0.086	0.150
R_postcentral_thickavg	0.090	0.268
L_lingual_thickavg	0.092	0.190
L_superiortemporal_thickavg	0.094	0.136
L_superiorparietal_thickavg	0.102	0.189
R_lateraloccipital_thickavg	0.104	0.226
L_cuneus_thickavg	0.106	0.198
L_paracentral_thickavg	0.108	0.190
L_fusiform_thickavg	0.115	0.116
R_paracentral_thickavg	0.130	0.169
L_postcentral_thickavg	0.133	0.139
L_pericalcarine_thickavg	0.151	0.091
L_inferiortemporal_thickavg	0.177	0.038
L_rostralmiddlefrontal_thickavg	0.188	0.059
L_precentral_thickavg	0.195	0.040
R_parsopercularis_thickavg	0.208	0.069

References

1. van Erp, T. G. M. *et al.* Cortical Brain Abnormalities in 4474 Individuals With Schizophrenia and 5098 Control Subjects via the Enhancing Neuro Imaging Genetics Through Meta Analysis (ENIGMA) Consortium. *Biol. Psychiatry* **84**, 644–654 (2018).
2. Larivière, S. *et al.* The ENIGMA Toolbox: multiscale neural contextualization of multisite neuroimaging datasets. *Nat Methods* **18**, 698–700 (2021).
3. Glasser, M. F. & Van Essen, D. C. Mapping human cortical areas in vivo based on myelin content as revealed by T1- and T2-weighted MRI. *J. Neurosci.* **31**, 11597–11616 (2011).
4. Van Essen, D. C. *et al.* The Human Connectome Project: a data acquisition perspective. *Neuroimage* **62**, 2222–2231 (2012).
